# Utility of Intravenous Curcumin Nanodelivery Systems for Improving *In Vivo* Pharmacokinetics and Anticancer Pharmacodynamics

**DOI:** 10.1021/acs.molpharmaceut.2c00455

**Published:** 2022-08-16

**Authors:** Mahsa Bagheri, Cornelus F. van Nostrum, Robbert Jan Kok, Gert Storm, Wim E. Hennink, Michal Heger

**Affiliations:** † Department of Pharmaceutics, Utrecht Institute for Pharmaceutical Sciences, Utrecht University, 3584 CG Utrecht, The Netherlands; ‡ Jiaxing Key Laboratory for Photonanomedicine and Experimental Therapeutics, Department of Pharmaceutics, College of Medicine, Jiaxing University, Jiaxing, Zhejiang 314001, PR China

**Keywords:** drug delivery, nanomedicine, micelles, nanoparticles, absorption, distribution, metabolism, excretion, cancer therapy

## Abstract

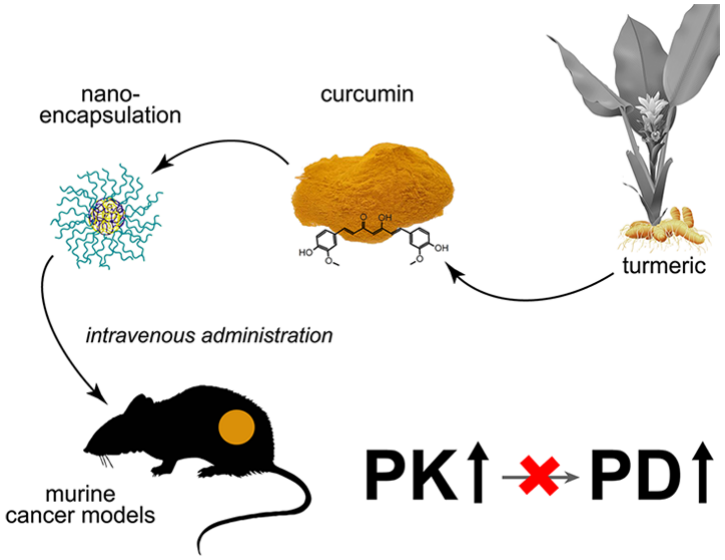

Curcumin nanoformulations for intravenous injection have been developed to offset poor absorption, biotransformation, degradation, and excessive clearance associated with parenteral delivery. This review investigates (1) whether intravenous nanoformulations improve curcumin pharmacokinetics (PK) and (2) whether improved PK yields greater therapeutic efficacy. Standard PK parameters (measured maximum concentration [*C*_max_], area under the curve [AUC], distribution volume [*V*_d_], and clearance [CL]) of intravenously administered free curcumin in mice and rats were sourced from literature and compared to curcumin formulated in nanoparticles, micelles, and liposomes. The studies that also featured analysis of pharmacodynamics (PD) in murine cancer models were used to determine whether improved PK of nanoencapsulated curcumin resulted in improved PD. The distribution and clearance of free and nanoformulated curcumin were very fast, typically accounting for >80% curcumin elimination from plasma within 60 min. Case-matched analysis demonstrated that curcumin nanoencapsulation generally improved curcumin PK in terms of measured *C*_max_ (*n* = 27) and AUC (*n* = 33), and to a lesser extent *V*_d_ and CL. However, when the data were unpaired and clustered for comparative analysis, only 5 out of the 12 analyzed nanoformulations maintained a higher relative curcumin concentration in plasma over time compared to free curcumin. Quantitative analysis of the mean plasma concentration of free curcumin versus nanoformulated curcumin did not reveal an overall marked improvement in curcumin PK. No correlation was found between PK and PD, suggesting that augmentation of the systemic presence of curcumin does not necessarily lead to greater therapeutic efficacy.

## 1 Introduction

**Figure 1:**
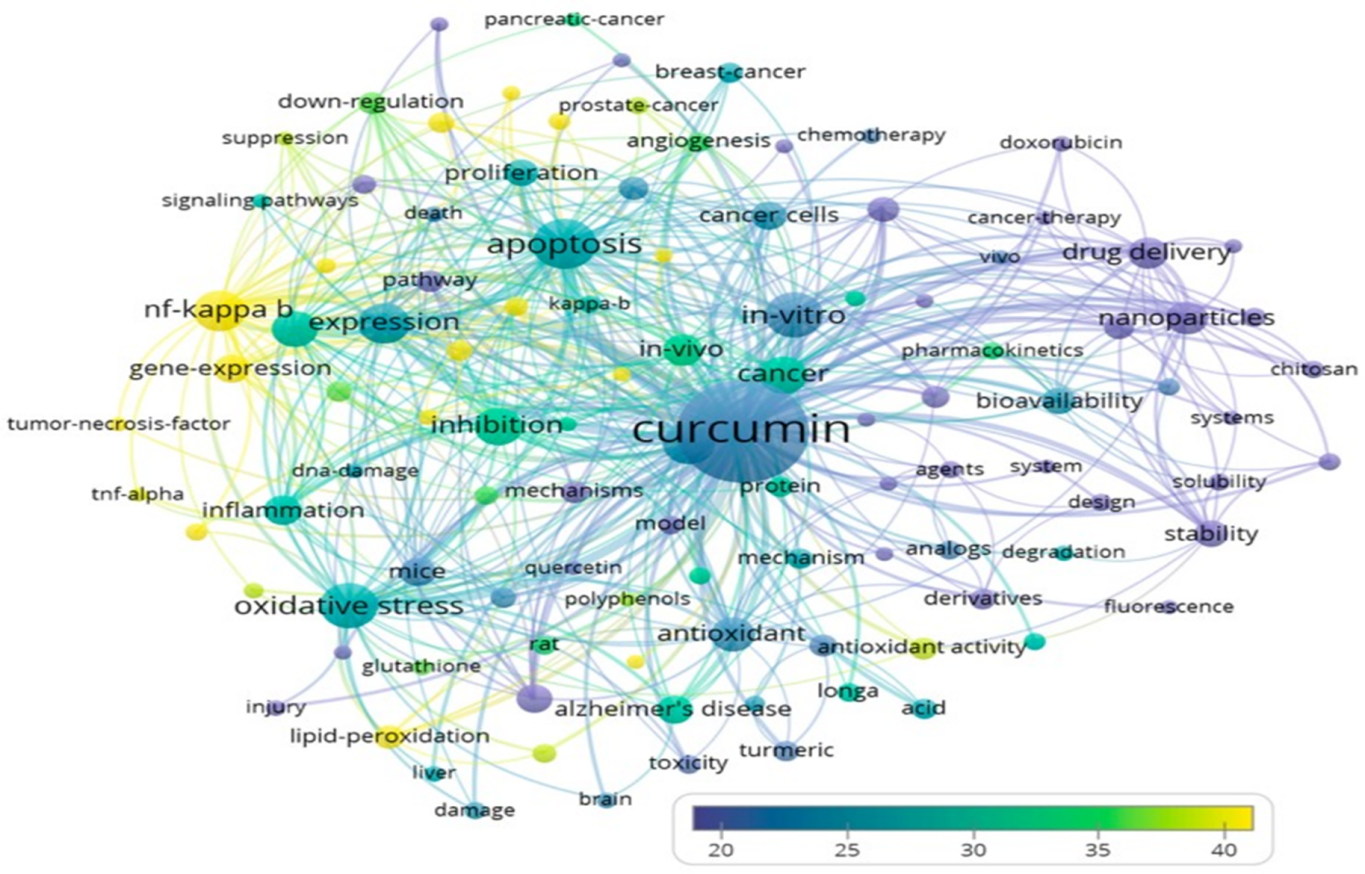
Bubble map of thematic keywords assembled from 18,036 publications on curcumin, showing most profound research interest in chronic disease fields that encompass oxidative stress, inflammation, and cancer as well as drug delivery systems and nanotechnology. Artwork reproduced from ref (10). Copyright 2019 without further modification and with permission under MDPI’s open access Creative Common CC BY license (https://www.mdpi.com/1420-3049/24/7/1393, https://www.mdpi.com/openaccess, and https://creativecommons.org/licenses/by/4.0/).

Curcumin is a polyphenolic phytochemical derived from the rhizome of *Curcuma longa*. The crude root (turmeric) traditionally serves as a spice and dietary supplement.^1,2^ Curcumin, the principal bioactive constituent in turmeric, is considered for the prevention and treatment of numerous diseases and conditions owing to its advantageous pharmacological properties^3−7^ and clinical safety profile.^8,9^ The complete curcumin research spectrum is presented in Figure 1. Curcumin-related research has drastically intensified over the past decade, attesting to its widely perceived potential utility as an active pharmaceutical ingredient.

Curcumin is investigated as a cancer therapeutic in light of its apoptosis-inducing effects in hyperproliferative cells.^11−14^ However, the compound is pharmacodynamically (PD) fierce but pharmacokinetically (PK) weak when it comes to treatment of cancer.^6^ Curcumin inhibits more than 40 vital metabolic pathways in malignant cells as a result of pleiotropic interactions with biomolecules, ultimately causing apoptotic cell death.^15^ Non-to-low proliferative healthy cells remain largely unafflicted.^16−18^ Although proof-of-concept regarding curcumin’s anticancer effects has been abundantly provided in mouse models of various types of human cancer,^19,20^ no notable therapeutic benefits have materialized in clinically approved and applied formulations.^6,21^ Two main reasons lie at the basis of this disconnect between animal research and clinical trials. First, many of the animal studies have been focused on systemically injected curcumin, while the vast majority of clinical studies hitherto have been conducted with orally dosed curcumin. Oral dosing gives rise to the second reason, which is that oral curcumin is associated with extremely low bioavailability^9,22^ due to poor intestinal absorption, extensive first-pass metabolism (phases I–III in enterocytes), degradation in pH-neutral aqueous medium, and ample liver metabolism (phases I–III in hepatocytes).^6^ Consequently, the plasma levels achieved with intravenous infusion—those that account for the favorable PD in animals—can never be reached in the circulation with orally administered curcumin. Orally dosed curcumin is therefore less favorable against cancer in humans than it is for nononcological indications.^7^

Considerable efforts have been invested in improving the PK profile of curcumin to augment PD in humans, including chemical modifications,^23,24^ coadministration with P-glycoprotein inhibitors such as piperine,^25−27^ and first pass circumventive approaches that involve encapsulation into nanoparticulate delivery systems for intravenous administration.^28−31^ The nanoencapsulation strategy has been instrumental in improving the PK of different hydrophobic drugs,^32−35^ of which several have been approved for clinical use by regulatory agencies.^36^ Nanoencapsulation of curcumin stabilizes curcumin in aqueous solution^37−39^ and solubilizes the compound. Nanoformulations for curcumin hence warrant close scrutiny, especially given the previous clinical successes with other nanoformulated drugs.

This critical appraisal paper therefore addresses the following questions: (1) Does intravenously administered nanoformulated curcumin improve PK compared to intravenously administered free curcumin; (2) which compositional attributes of the nanoformulations are responsible for the improvement in PK; and (3) do improved PK profiles translate to improved PD in murine models of human cancer?

## 2 Data Curation and Analysis

Readers should note that references to Supporting Information are indicated with prefix ‘S’.

PK data were collected from mouse and rat studies in which intravenously administered curcumin nanoformulations were compared to free curcumin. Plasma extraction and curcumin quantification methods differed between the studies. Extraction was chiefly performed by precipitation of plasma proteins with water-miscible organic solvents (e.g., acetonitrile and methanol)^40−55^ or liquid phase extraction with ethyl acetate.^56−64^ Curcumin was quantified by absorbance, fluorescence, or mass spectrometry. Almost all studies employed a chromatography system coupled to a spectroscopic detector, while a handful of studies used a cuvette-based or plate reader spectrometer.^41,46,49,52,53^

For the reproduction of curcumin plasma concentration–time curves, mouse and rat studies were selected where the PK of free curcumin (*n* = 15) and nanoencapsulated curcumin (*n* = 15) could be relatively accurately extrapolated from the respective figure. The figures were imported into Adobe Photoshop from the PDF version of the publication at 600 dpi resolution, and lines were protracted from the data point to the *y*-axis and *x*-axis to estimate the curcumin concentration at a given time point, respectively. Time points were verified by cross-referencing the methods section where available. Only studies were included where the first plasma level measurement was performed within the first 5 min after injection because of curcumin’s relatively fast elimination kinetics as pointed out in this paper. Data were normalized to the plasma concentration at the earliest time point and plotted in GraphPad Prism (GraphPad Software, San Diego, CA, USA). The data normalization allowed interstudy comparison of both free and nanoencapsulated curcumin. Moreover, the data serve as a standard for the validation of study models (especially for the free curcumin controls) and enable the benchmarking of curcumin nanoformulations to gauge their utility. Normalization to the plasma concentration measured several min after intravenous administration introduced some inaccuracy (i.e., overestimation) of the fraction of residual curcumin in the circulation that is equal to the “loss” of plasma curcumin during the time from injection to first measurement. This phenomenon only slightly impacts the amplitude but not the trend of the curve, which was predicated on actual plasma concentrations. When comparative analyses are performed, the vertical skewing of readouts could be minimized through protocol standardization (i.e., the time of first measurement is <5 min after curcumin injection).

In a separate analysis, normalized plasma concentrations of free curcumin and nanoencapsulated curcumin were fitted with a two-phase decay fit function to reflect distribution (KFast segment of the curve) and clearance (KSlow segment of the curve), corresponding to a two-compartment PK model. Fitting was performed on the entire measurement interval, which in some studies extended to 24 h postinjection. Given the rapid decay in curcumin plasma concentration, only the first 4 h postinjection is presented. It should be noted that these distribution and clearance phases theoretically represent a superimposed mixture of singular phases of the nanoparticles carrying the curcumin and the free curcumin that has exited the nanoparticle. Nonetheless, the singular phases of the curcumin nanoformulations were not parsed given their comparable pattern to free curcumin, indicating that the curcumin exited the nanoparticles during the first 5 min after injection and subsequently behaved as free curcumin (with the exception of a few formulations that better retained the curcumin cargo). Eleven of the 15 studies (73%) on free curcumin conformed to this model and yielded a goodness of fit (*R*^2^) value of ≥0.9970, whereas 12 of the 16 studies (73%) on nanoencapsulated curcumin yielded an *R*^2^ value of ≥0.9912. Finally, for comparative analysis, the normalized plasma concentrations of curcumin and nanoencapsulated curcumin were averaged per time point and the means ± SD were plotted. The data points were fitted with a two-phase decay fit function.

The plasma curcumin concentration over time is typically analyzed by noncompartmental and compartmental models (section S2, Figures S1–S3). Among the common PK parameters, the maximum concentration (*C*_max_), area under the curve (AUC), and elimination half-life (*t*_1/2_) are frequently reported. For purposes of simplicity, the *C*_max_ values reported in this paper reflect the highest plasma concentration of curcumin at the earliest measured time point (1–15 min) and are therefore referred to as ‘measured *C*_max_.’ In our analysis, the *C*_max_ definition therefore differs from the conventional definition used in the context of orally administered drugs. Conversely, only a few studies reported clearance (CL) and distribution volume (*V*_d_), even though CL and *V*_d_ can be calculated from the plasma concentration–time curve.^65^ In combination with AUC and *t*_1/2_, these parameters indicate how quickly a compound is eliminated and reflect the propensity of the drug to stay in the circulation or distribute to other compartments.^66^ The CL and *V*_d_ were therefore calculated using the data available for AUC and *t*_1/2_ in instances where CL and *V*_d_ were not reported (see section S2 and Figure S4 for more detailed information). The *V*_d_ values were calculated when the values were not explicitly reported in the included studies. To enable interstudy comparative analysis of the PK parameters, values were converted to harmonize the units and the AUCs were subsequently normalized to the administered curcumin dose and expressed as (μg·h/L)/(mg/kg). Readers should not that the definition of *V*_d_ for nanoencapsulated drugs may veer from the classical definition of *V*_d_ for drugs whose plasma data follow log–linear decay. Inasmuch as the curcumin concentration kinetics curves did not fundamentally differ between free curcumin and nanoencapsulated curcumin (with the exception of 5 nanoformulations), and as this parameter was chiefly used as a predicate for investigations on the PK–PD relationship, this technical difference in *V*_d_ definitions was acknowledged but discounted from the analyses. Semantics related to *V*_d_ did not distort the main conclusions.

Finally, the correlation between PK and PD was analyzed in GraphPad Prism. Specifically, the correlation between the nominal difference in the percentage of tumor growth inhibition (%TGI, *y*-axis variable) and (1) the AUC, (2) the administered dosage, and (3) the nanoformulated curcumin:free curcumin AUC ratio (*x*-axis variables) was determined. The difference in %TGI was stratified into nanoformulated curcumin versus control, free curcumin versus control, and nanoformulated curcumin versus free curcumin using the % TGI values as measured at the end of the experiment as input data (Figure S5). The actual %TGI was extrapolated from the respective figure or derived from the text. The data obtained from mouse and rat studies were clustered. Accordingly, the nominal difference in %TGI ranges from 0 to 100%, where 0% means that the intervention had no tumoricidal effect compared to the control group (buffer or empty carrier in case of nanoformulated/free curcumin comparisons to control, and free curcumin when comparing nanoformulated versus free curcumin). Similarly, a value of 100% means that the tumor had been completely eliminated at the end of the experiment. However, complete eradication or regression of tumors was not observed in any study. Correlation analysis for the first two variables (AUC and dosage) is straightforward and spurs the expectation that, given the anticancer properties of curcumin and the improved PK of nanoformulated curcumin, a positive relationship exists between AUC and dosage versus the nominal difference in %TGI. The correlation between the nominal difference in %TGI and the nanoformulated curcumin:free curcumin AUC ratio entailed a slightly different rationale. Studies were included where the curcumin nanoformulation AUC was greater than the AUC of the free curcumin control (i.e., nanoformulated curcumin:free curcumin ratio of >1), and where respective controls had been properly implemented in the experimental design. Subsequently, the nominal difference in %TGI was plotted of the nanoformulated curcumin versus vehicle/solvent control and of the free curcumin versus solvent control as a function of the nanoformulated curcumin:free curcumin AUC ratio. These data provided insight into the level of antitumor activity per degree of improved PK due to curcumin nanoencapsulation to assess the expectation that a positive correlation would be observed.

## 3 Nanoformulations Improve Multiple Curcumin Pharmacokinetics Parameters Compared to Nonformulated Free Curcumin

### 3.1 Pharmacokinetics of Intravenously Administered Free Curcumin

**Figure 2:**
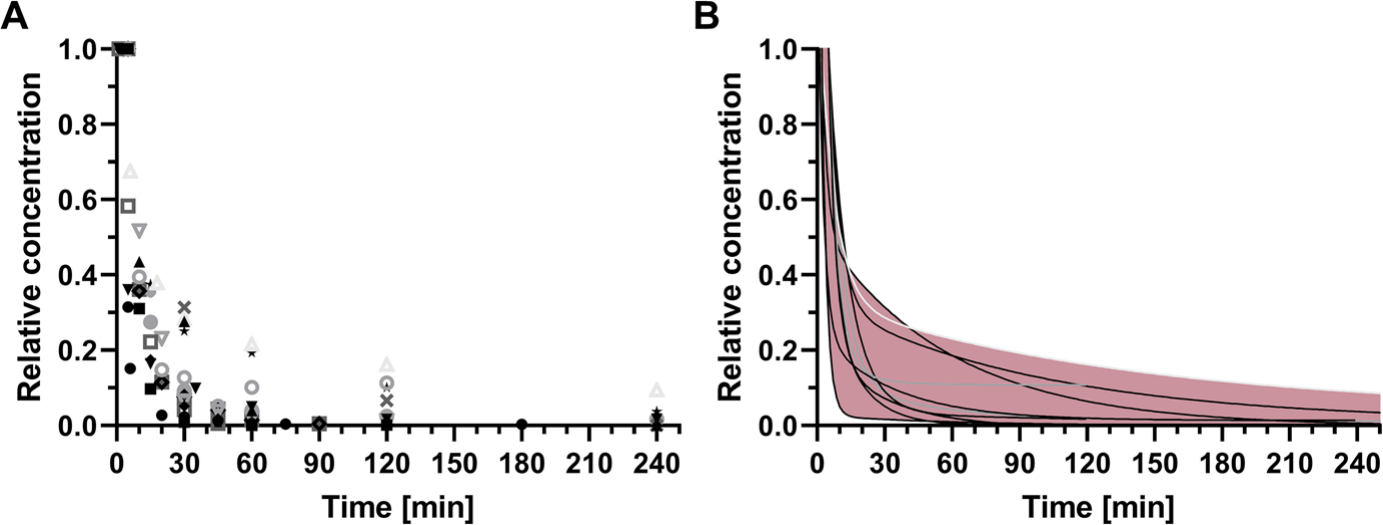
PK of free curcumin following intravenous administration in mice and rats as reported in literature. The *x*-axis and *y*-axis data were extracted from figures in published papers and verified by cross-referencing the text (where available). Plasma concentrations were normalized to the concentration measured at the earliest time point, not exceeding an interval of 5 min between injection and measurement. Normalized concentrations are provided in (A) as a function of circulation time. Data were compiled from 15 studies.^43,48,49,51,53,54,56,60,61,63,64,70−73^ The points were fitted with a two-phase decay fit function to reflect distribution (fast phase) and clearance (slow phase) (B). Eleven studies conformed to this PK model (*R*^2^ ≥ 0.9970).^43,49,51,53,54,56,60,61,64,72,73^ The maximum and minimum concentrations are represented by the outer bounds of the 11 fits (pink region). Compartmental deflection generally occurred between 20 and 30 min after intravenous administration.

Representative PK profiles of free curcumin in mice and rats are depicted in Figure 2. Plasma concentrations followed a biphasic pattern that is characterized by a rapid decay due to biodistribution and a slower decay due to elimination (Figure S1). More than 50% of the injected dose was no longer retrievable from plasma 10 min after administration, suggesting rapid tissue distribution. Curcumin is known to distribute to multiple organs, including the liver, kidneys, lungs, spleen, and brain^42,49,56^ and undergoes renal and hepatobiliary clearance.^42,67−69^ The switch from tissue distribution as the dominant cause of plasma decay to mainly clearance typically occurred between 20 and 30 min postinjection. At 30 min, only 13 ± 10% (mean ± SD, *n* = 14) of the injected dose remained in the circulation and gradually dissipated during the subsequent 3–4 h.

Two key considerations should be pointed out in case of free curcumin. First, interspecies differences notwithstanding, the administered dose, type of solvent/vehicle, experimental design, and analytical method may differentially affect PK parameters.^74^ This is illustrated by the rather wide relative concentration range per time point as presented in Figure 2. Some of the solvents/solubilizers that were used are micelle-forming surfactants (such as Kolliphore and Tween) that may prolong the systemic presence of curcumin. The consequences of curcumin solubilization by these excipients before or after intravenous administration on PK are further elaborated in section S3.1. Also, the possibility of assay interference should be taken into account since some studies did not use chromatography-based equipment for effective compound separation. Second, with the therapeutic efficacy of intravenously administered free curcumin being relatively low (section 4), it is not difficult to fathom how therapeutically impotent orally dosed curcumin is in oncological patients. Bioavailability and therefore systemic concentrations are significantly hampered by the aforementioned absorption and metabolism issues (section 1) and ultimately yield systemic concentrations that are pharmacologically moot in an oncotherapeutic setting. These concerns have already been addressed for curcumin.^75^ Nevertheless, for certain nononcological indications (such as systematic inflammation, oxidative stress, etc.), the achieved plasma levels of oral curcumin are clinically adequate.^7^

### 3.2 Nanoencapsulation of Curcumin Improves the Measured *C*_max_ in the Distribution Phase

For the analysis of measured *C*_max_ (defined in section 2), 27 studies were included in which free and nanoencapsulated curcumin were administered intravenously into mice and rats at equal curcumin doses. For intravenously administered compounds, the measured *C*_max_ corresponds to the highest concentration of compound in plasma detected immediately after injection, and in theory approximates the injected dose per mL blood.

**Figure 3:**
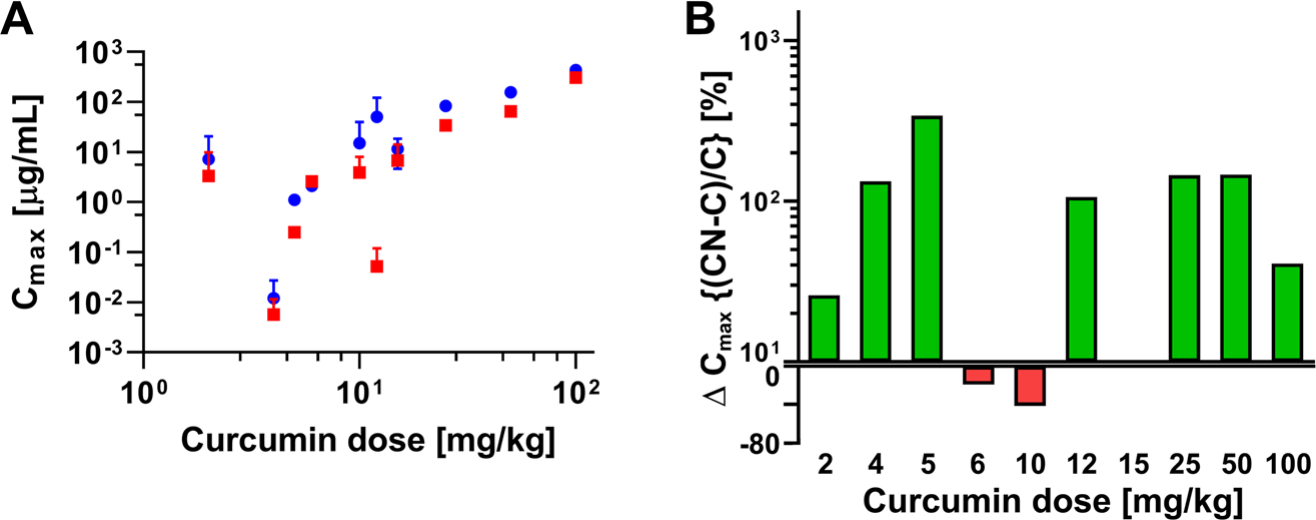
(A) Mean ± SD measured *C*_max_ of free curcumin (red square) and curcumin nanoformulations (blue circle) plotted as a function of injected dose in mice and rats (*n* = 27). (B) Fold-increase (green bars, log scale) and fold-decrease (red bars, linear scale) in the measured *C*_max_ of nanoencapsulated curcumin relative to the measured *C*_max_ of free curcumin, plotted as a percentage and as a function of injected curcumin dose. Abbreviations: Δ, delta (change); CN, curcumin nanoformulation; C, free curcumin.

As shown in Figure 2, the steep distribution phase of free curcumin typically lasts 20 min, followed by deflection into the shallower clearance phase. The range of the injection-measurement intervals was 1–15 min for the included studies.^40−49,51−54,56,58−64,70−73,76−80^ The measured *C*_max_ values, stratified by injected dose, are presented in Figure 3A. The selected time frame allowed for the detection of PK differences between free curcumin and nanoencapsulated curcumin in the distribution phase only.

In line with expectations, the measured *C*_max_ of curcumin increased with injected dose for both free and nanoformulated forms. However, the injected dose-measured *C*_max_ relationship for nanoencapsulated curcumin (Spearman’s ρ = 0.909; *p* ≤ 0.001) showed a stronger correlation compared to the free form (Spearman’s ρ = 0.793; *p* ≤ 0.01) (Figure 3A). This dichotomy suggests that free curcumin exits the plasma compartment more profusely during the distribution phase than the curcumin contained in the nanoparticulate delivery systems. Of the 27 studies, the majority (*n* = 19) yielded a higher measured *C*_max_ for the nanoformulations compared to the respective free form (Figure 3B). The increase in measured *C*_max_ was around 100% for most dose comparisons. The data indicate that formulating curcumin into nanocarriers generally improves the measured *C*_max_ and hence potential exposure of the tissues to the phytochemical compound (whether still encapsulated or released from the nanocarrier) during the distribution phase. Nevertheless, this is not a rule for every type of nanoparticulate carrier and the differential exposure also depends on the time interval between the injection and the first measurement time point, the curcumin release rate from the carrier, as well as the ability of the nanoparticles to extravasate and deliver the cargo into tumor cells such that cytotoxicity is conferred.

Currently it is not clear why the injected dose-measured *C*_max_ correlation is stronger for nanoformulations than for the free form and why nanoencapsulation improves the measured *C*_max_ so considerably. The most plausible reason is that free curcumin rapidly settles into the membranes of blood cells upon entry into the systemic circulation,^81^ owing in part to its log *P* of 2.5.^82^ The mechanistic details that underlie curcumin-membrane interactions are provided elsewhere.^83−85^ This fraction of blood is not included in the plasma analysis, which does not apply to the cell-unassociated nanoparticulate curcumin that remains in the plasma fraction during sample processing. Another possibility is that hepatic and renal clearance already contribute to the concentration decline in the distribution phase and that the clearance favors free curcumin due to steric factors in terms of particle size relative to the size of the endothelial fenestrations in the kidneys and liver. Fast biliary clearance of free curcumin after intravenous injection, evidenced by the detection of curcumin in bile as early as 5 min after intravenous administration, was observed in rats.^86^ Finally, free curcumin is more amenable to degradation in plasma than nanoencapsulated curcumin, where the excipient encapsulating curcumin offers protection chemically and/or sterically,^37,87,88^ culminating in comparatively lower retrieval of free curcumin from plasma. Hitherto no studies elaborately assessed the degradation rate of curcumin in a plasma matrix, and therefore the extent of this effect on the PK of curcumin is still unknown. It should be noted that, as was recently also demonstrated by our group,^55^ most nanoparticulate curcumin carriers act as solubilizers and do not firmly retain the curcumin in the nanoparticle following systemic administration. Clearance of nanoparticulate curcumin is also quite steep during the distribution phase,^43,48,49,51,53,54,56,60,61,63,64,70−73,76^ albeit more delayed compared to free curcumin probably due to gradual release of curcumin from the nanoparticles.

### 3.3 Curcumin AUC Is Improved by Nanoencapsulation, but Not with Every Formulation Type

The AUC signifies a biological system’s comprehensive exposure to a drug and, when juxtaposed to the PK curve (Figure 2), gives insights into the clearance rate of the drug. This parameter is instrumental in the analysis of different formulations in terms of their extent of drug exposure when administered at the same dose.^89^ Curcumin is degraded in plasma^90^ and rapidly removed from the circulation via renal and hepatic clearance^42,67,69,86^ and accumulation in various organs.^91^ This, together with the fact that curcumin is further metabolized and degraded in target cells,^92,93^ accounts for relatively brief PD activity after intravenous administration and accumulation in target tissue. Extending the circulatory presence (i.e., AUC) of curcumin by nanoencapsulation may therefore benefit PD efficacy. Moreover, the AUC is a better measure for pharmacological potency of the active principal than the measured *C*_max_, particularly for intravenously administered drugs that are rapidly removed from the plasma compartment.

**Figure 4:**
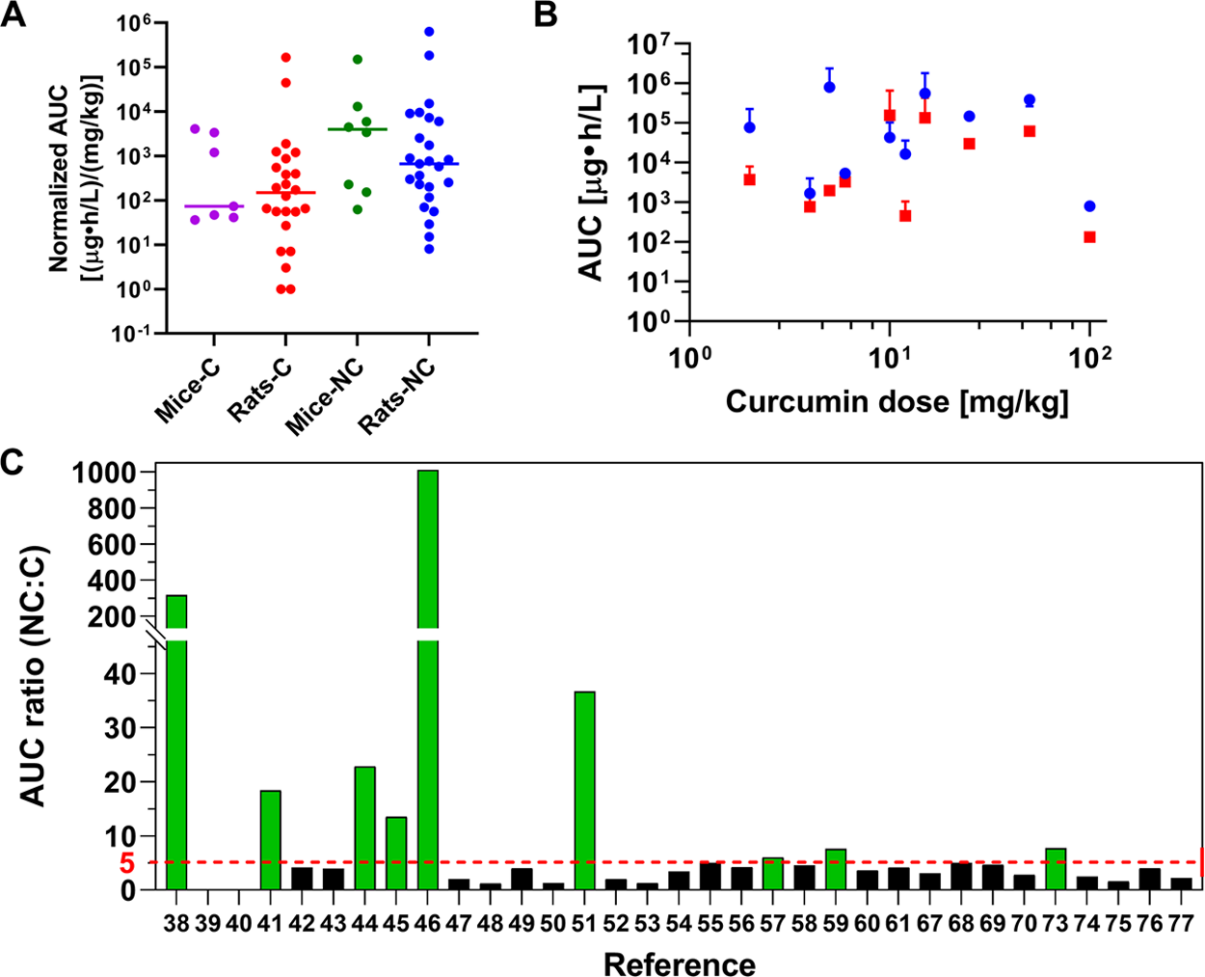
(A) Normalized AUC of free curcumin (C) and curcumin nanoformulations (NC) in mice and rats. Data were normalized to the injected dose. The horizontal line indicates the median. There is a significant difference between the normalized AUC values between the C and NC group in mice (*P* = 0.031) and rats (*P* = 0.026) (Mann–Whitney U test). (B) AUC of free curcumin (red square) and curcumin nanoformulations (blue circle) as a function of injected dose. (C) The AUC ratio of nanoformulated curcumin (NC) versus free curcumin (C) plotted per study in mice and rats. The dotted line represents a cutoff at an AUC ratio of ≥5. Studies reporting an NC:C AUC ratio of ≥5 are indicated in green. No data were available for AUC of free curcumin in refs (41, 42, and 55).

The AUC values of free and nanoencapsulated curcumin were derived from published studies in mice and rats. The AUC values were reported either as AUC_0-*t*_^40,43,44,51,53,58,61,71^ or AUC_0-infinity_.^45−50,52,55−57,59,60,62−64,70,72,73,77−80^ Some studies did not specify the AUC reporting method.^41,42,54,76^ The AUCs were normalized to the injected dose (Tables S1–S4) and plotted (Figure 4A). Table 1 summarizes the range of normalized AUC values for free curcumin and curcumin nanoformulations and provides the median and mean of the clustered data in both mice and rats. Notwithstanding the wide spread of normalized AUC values, a statistically significant difference was found between free versus nanoencapsulated curcumin AUC.

**Table 1:** Descriptive Statistics of Normalized AUC (μg·h/L)/(mg/kg) of Free Curcumin and Curcumin Nanoformulations in Mice and Rats^a^

	free curcumin		curcumin nanoformulations	
	mice	rats	mice	rats
number of studies	7	23	9	24
minimum	36	1	62	8
maximum	4,075	167,000	149,705	632,000
median	73	171	4,482	714
mean	1,260	9,532	20,360	36,402
SEM	658	7,411	16,225	26,996

a Abbreviation: SEM, standard error of the mean. The circulating blood volume is 78–80 mL/kg in mice and 50–70 mL/kg in rats.^94^ Data assembled from refs (40−49, 51−64, 70−73, and 76−80). Statistical analysis of normalized AUC values between free curcumin and nanoencapsulated curcumin yielded a significant difference in mice (*P* = 0.031) and rats (*P* = 0.026); Mann–Whitney U test.

To determine whether data clustering, where dosing was discounted as variable, resulted in a misrepresentative AUC comparison, the AUC values were replotted as a function of the injected dose (Figure 4B). At all but one dosage the nanoformulated curcumin outperformed the free curcumin in terms of AUC. Also, the majority (22/31; 64%) of the studies revealed that curcumin nanoencapsulation augmented the AUC by a factor 1.3–5 compared to free curcumin (Figure 4C). Accordingly, the AUC improvements achieved by nanoencapsulation become masked in the normalized clustered data (Figure 4B and C versus A). It also becomes clear that the type of nanoformulation plays a role in the extent of AUC improvement, addressed further in section 3.5.

**Figure 5:**
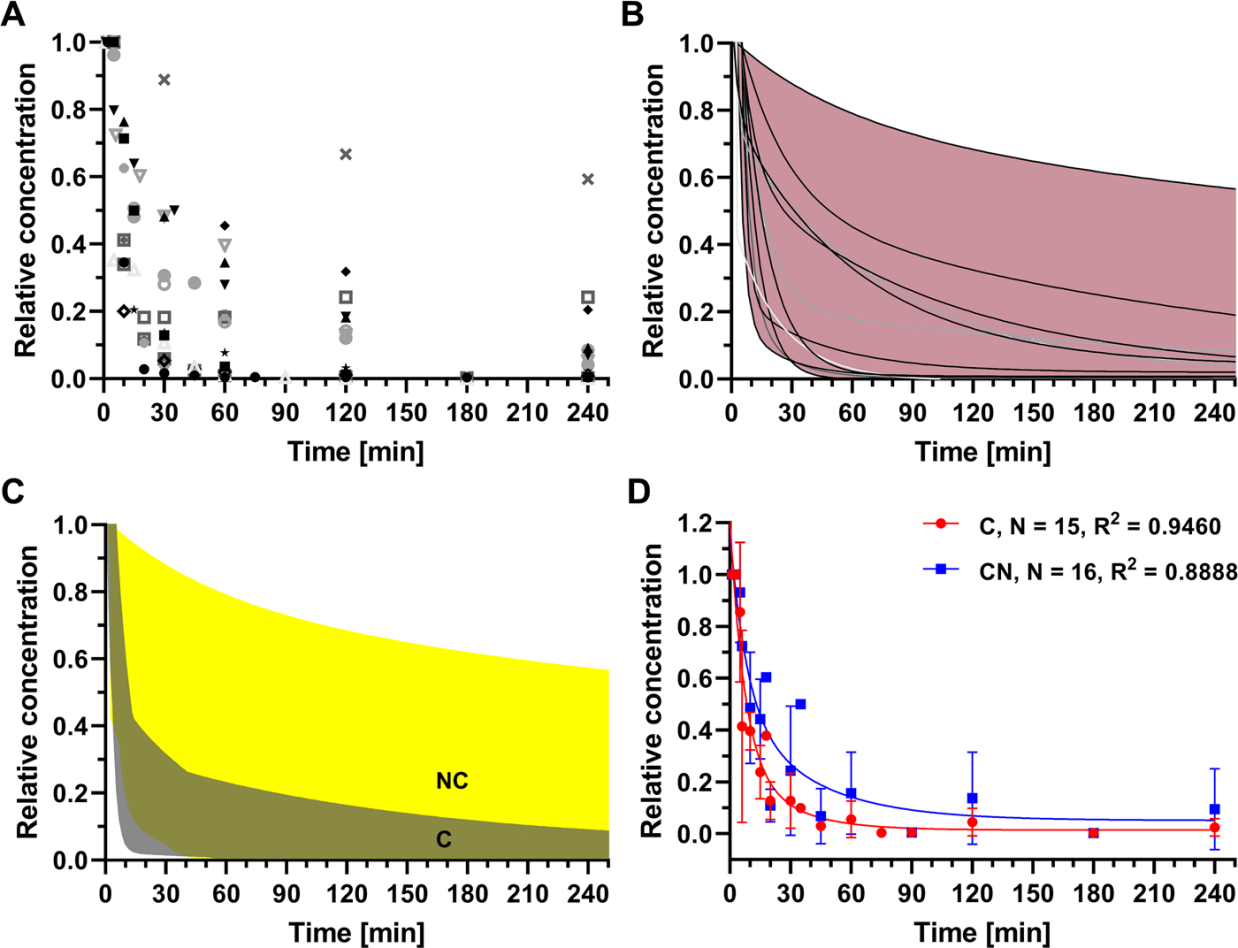
PK of nanoencapsulated curcumin following intravenous administration in mice and rats as reported in literature. The *x*-axis and *y*-axis data were extracted from published figures and verified by cross-referencing the text. Plasma concentrations were normalized to the concentration measured at the earliest time point, not exceeding an interval of 5 min between injection and measurement. Normalized concentrations are provided in (A) as a function of time after intravenous injection. Data were compiled from 15 studies.^43,48,49,51,53,54,56,60,61,63,64,70−73^ The points were fitted with a two-phase decay fit function to reflect distribution (fast phase) and clearance (slow phase) (B). Twelve studies conformed to this PK model (*R*^2^ ≥ 0.9912).^43,48,49,51,53,60,63,64,70−73^ The maximum and minimum concentration are represented by the outer bounds of the 12 fits (pink region). For some of the nanoformulations, compartmental deflection occurred between 10 and 30 min following intravenous administration. However, 6 formulations exhibited deflection points at >30 min after intravenous administration. The temporal spread in nanoformulated curcumin concentration (NC; B) was superimposed on that of free curcumin (C; Figure 2B) to show the pharmacokinetic and potential pharmacodynamic gain offered by curcumin nanoencapsulation (C), which applies mainly to the formulations where the traces in (B) course above the dark gray region designated as ‘C’ (formulation 1,^60^ formulation 5,^43^ and formulation 7^53^ in Table 2 and refs (49 and 71)). The relative plasma concentrations of free curcumin (Figure 2A) and nanoencapsulated curcumin (A) were averaged per time point and plotted as mean ± SD as a function of time (D). The mean values were fitted with a two-phase decay fit function. The sample size and goodness of fit (*R*^2^) values are reported in the top right.

As was performed for free curcumin (Figure 2), time–plasma concentration curves for nanoencapsulated curcumin were recreated from the published figures and cross-referenced with the text. The data are presented in Figure 5A,B and demonstrate that a substantial portion of curcumin nanoformulations was not effective in raising the relative plasma concentration of curcumin when compared to the free curcumin counterpart (Figure 2A). However, several formulations were able to protract the distribution → clearance deflection point beyond the 30 min threshold (*n* = 6) that was observed for free curcumin and maintain higher plasma levels of curcumin (*n* = 5). The 5 formulations exhibit two-phase decay traces that reside above the upper boundary of the kinetics traces of free curcumin (Figure 3B,C). Despite the 5 well-performing formulations, the overall average relative curcumin plasma concentration of the nanoformulations does not convincingly differ from that of free curcumin (Figure 5D), underscoring the fact that raising the AUC of curcumin and hence its potential PD efficacy relies heavily on formulation type.

In our recently published study on curcumin-loaded polymeric micelles composed of poly(ethylene glycol)-*b*-poly(*N*-2-benzoyloxypropyl methacrylamide) (mPEG-*b*-p(HPMA-Bz)), we observed a notable incongruence between the PK of the carrier versus curcumin (see Figure 9 in ref (55)). Twenty-four hours after intravenous administration, approximately 50% of the Cy-7-labeled mPEG-*b*-p(HPMA-Bz) micelles was still present in the mouse circulation, whereas 90% of the loaded curcumin had been eliminated from the plasma compartment at 1 h postinjection. The curcumin elimination from plasma was likely facilitated by curcumin exiting the nanocarrier.

This brings about the following important matter: It is crucial to understand the underlying process of clearance of nanoformulated curcumin from plasma, which can stem from the systemic removal of the curcumin-containing nanocarrier as an intact drug-nanocarrier entity or from drug-nanocarrier destabilization in blood, causing release of the loaded curcumin and subsequent clearance of the released curcumin independently of the nanocarrier. The kinetics curves of curcumin nanoformulations presented in Figure 5B that resemble those of free curcumin in Figure 2B may reflect the latter process. A mechanistic understanding of the PK of nanoencapsulated curcumin is critical to appreciate the impact of nanocarriers on PD efficacy of curcumin inasmuch as two distinct scenarios are likely to happen. The unstable curcumin nanoformulation with comparable PK profile to that of free curcumin could either emulate the PD efficacy of free curcumin (rendering the nanocarrier nonadditive from a therapeutic standpoint) or improve the PD efficacy compared to the free curcumin. The latter phenomenon echoes other unstable nanoformulations such as Genexol and Abraxane. These nanoformulations exhibit a PK profile that is similar to nonencapsulated paclitaxel but yield superior therapeutic efficacy.^95−97^

### 3.4 The Distribution Volume Is Positively Affected by Nanoencapsulation, but Depends on Formulation Type

The elimination of curcumin from plasma over time can have several causes. In addition to curcumin ending up in the discarded infranatant during sample processing (section 3.2), it may be metabolized and/or degraded,^90,98^ excreted renally and metabolized hepatically,^42,67,69,86^ or diffuse into the extravascular space (i.e., distribution into tissues^91,99,100^). When considered in the context of oncopharmacology, the difference between renal/hepatic CL and tissue distribution is critical, whereby the former occurs at the detriment of PD efficacy. CL and distribution volume (*V*_d_) can be calculated (section S2). The *V*_d_ represents the ratio of the amount of drug present in the body (injected dose) to the concentration of drug measured in plasma and is expressed as the volume of fluid required to be present in the extravascular space for achieving the concentration to be equivalent to the plasma concentration. The *V*_d_ correlates positively with the amount of drug distributed into tissue, is directly proportional to the lipophilicity of a compound, and inversely proportional to the extent of plasma retention.^101^

**Figure 6:**
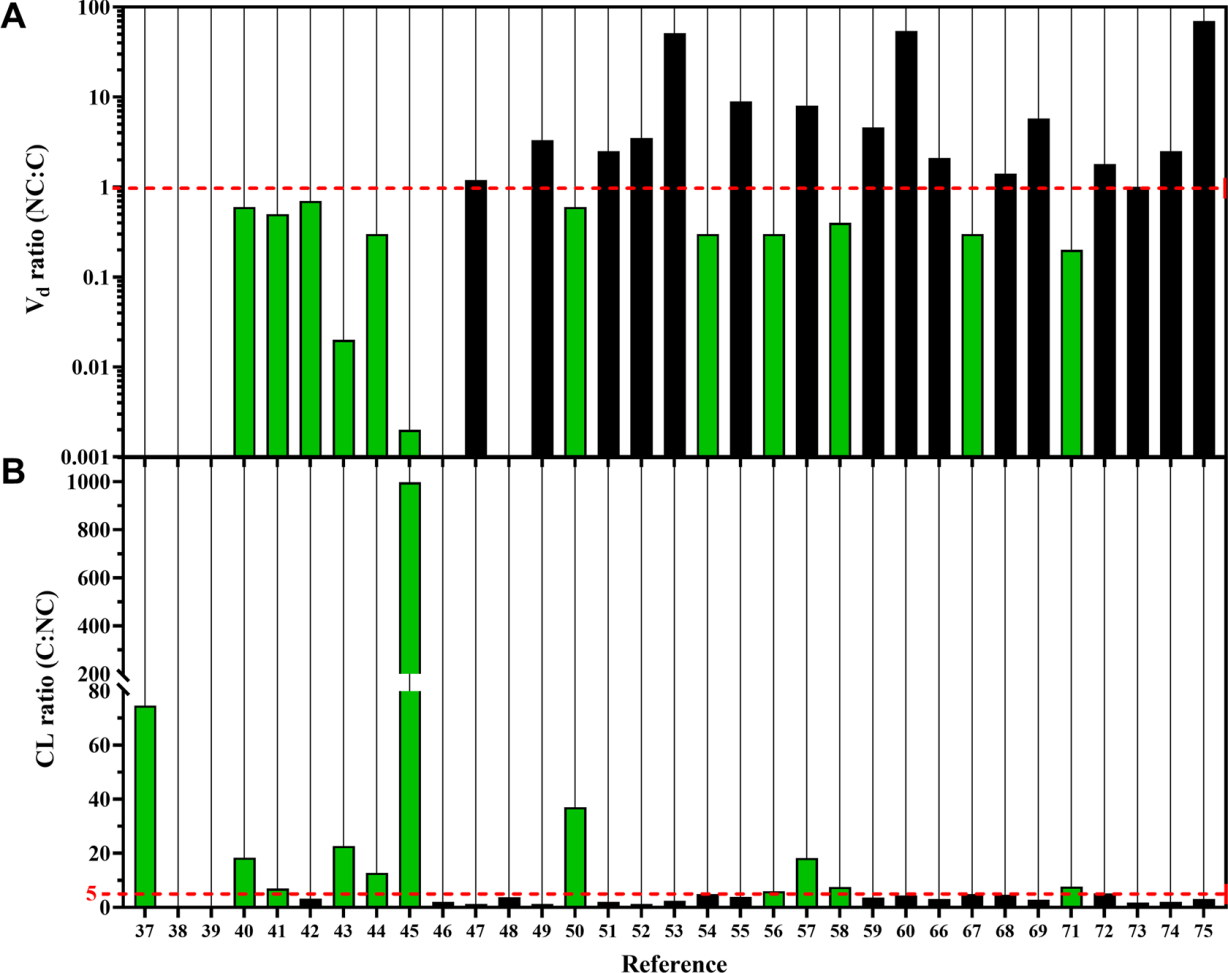
Distribution volume (*V*_d_) ratio (A) and clearance (CL) ratio (B) of curcumin nanoformulations (NC) in relation to experiment-matched free curcumin (C) in mice and rats. The *V*_d_ ratio could not be calculated for refs (40−42, 49, 51, and 55) due to insufficient data. An NC:C *V*_d_ ratio of <1 (green bars) and a C:NC CL ratio of >5 (green bars) indicate that the NC formulations were better retained in plasma than free curcumin. The dotted line in (B) represents a cutoff at an AUC ratio of ≥5. The references in (B, *x*-axis) are imposable on (A).

The *V*_d_ data are presented in Tables S1–S4. The calculated values, marked with an asterisk in Tables S1–S4, show the *V*_d_ at the terminal phase of elimination (schematically explained in Figure S1). The range of *V*_d_ values is very broad: 0.002–1,376 L/kg and 0.06–882 L/kg for free curcumin and nanoformulated curcumin, respectively. This spread can be due to variation in PK and differences in the *V*_d_ calculation method.^65^ Two outlier studies^57,64^ reported unrealistic *V*_d_ values (2 and 4 mL/kg for free curcumin) that are far below the average blood volume in mice and rats (50–80 mL/kg or 7–8% body weight),^102,103^ casting doubt about the reliability of data analysis. To provide perspective, the total body water:body weight ratio in mice is roughly 0.6 L/kg,^104^ while the total plasma volume is approximately 49 mL/kg and extracellular water content is 232 mL/kg.^105^ Additionally, the *V*_d_ of curcumin nanoformulations was lower,^43−48,53,58,60,62,71,76^ similar,^50,72,77,78^ or higher^52,54,56,57,59,61,63,64,70,73,79,80^ than free curcumin (Figure 6A). An NC:C *V*_d_ ratio of <1 indicates that curcumin was stably retained in the nanoparticles (and hence remained in the circulation), while a *V*_d_ of ≥1 suggests that the nanocarriers most likely acted as solubilizers with poor cargo retention, as addressed in section 3.3.

Duan et al.^57^ and Ma et al.^80^ observed a substantial increase in *V*_d_ for curcumin when loaded into chitosan/poly(butyl cyanoacrylate) nanoparticles and methoxy poly(ethylene oxide)-*block*-poly(ε-caprolactone) micelles, respectively. The authors postulated that this might be due to the sequestration of larger curcumin-loaded nanoparticles by cells of the reticuloendothelial system, which released curcumin and acted as a reservoir.

In regard to cancer treatment, a high *V*_d_ does not have to be detrimental to PD efficacy per se as long as the (nanoparticulate) curcumin predominantly accumulates in the target tissue to induce apoptosis of tumor cells. Exemplary proof-of-concept was recently provided by Rodell et al.,^106^ who designed cyclodextrin-based nanoparticles encapsulating Toll-like receptor small molecular agonists intended to target to tumor-associated macrophages to steer their polarization and improve cancer immunotherapy efficacy. The drug-bearing nanoparticles accumulated most profoundly in the tumor tissue compared to the other 11 noncancerous tissues measured, which resulted in superior tumoricidal effects versus the nonencapsulated drug, despite distribution into off-target tissues. This not only contextualizes the practical significance of the *V*_d_ but also underscores the importance of including experiments focused on curcumin biodistribution, or at least curcumin accumulation in target tissue. Moreover, a sizable *V*_d_ can potentially be offset with efficient tumor targeting tools conferred on the drug delivery system.^107^

CL represents the volume of plasma from which a substance is removed per unit time. As opposed to excretion, which strictly reflects the compound leaving the body through urine, feces, and sweat, CL is a measure of plasma disappearance rate and may encompass excretion when measurements are based solely on plasma levels. *V*_d_ and CL are closely related in that both metrics provide an indication of the amount of substance remaining in the circulation at or after a certain time. Accordingly, the C:NC CL ratios plotted in Figure 6B roughly reflect the *V*_d_ ratios portrayed in Figure 6A, attesting to the fact that 8 of 31 formulations^43,46−48,53,60,62,76^ were clearly capable of better retaining curcumin in the circulation and thereby increased the statistical probability that the encapsulated curcumin could reach and accumulate in a tumor exploiting the enhanced permeation and retention effect.^108,109^ The 9 nanoformulations that consistently outperformed free curcumin on the basis of AUC (Figure 4C), *V*_d_ (Figure 6A), and CL (Figure 6B) are highlighted in Table 2, with their compositional attributes and physicochemical characteristics discussed in the next section.

**Table 2:** Summary of the Physicochemical Properties of Intravenously Administered Curcumin Nanoformulations with an AUC Ratio of >5 Compared to Free Curcumin (NC:C)^a^

curcumin nanoformulation	mean size (nm)	mean ZP (mV)	loading capacity (%)	release profile	free curcumin vehicle	species	ID (mg/kg)	AUC ratio NC:C	ref
*Formulation 1*: mPEG-PCL^b^	27	NA	13.	54.6% of total curcumin release within 9 days in PBS + 0.5% Tween 80 at pH = 7.4	Cremophor EL and dehydrated alcohol (1:1, v/v)	SD rats	100	6.0	(60)
*Formulation 2*: mPEG-PLA^c^	70	2.9	4.8	85% curcumin release after 96 h in physiological saline containing 1% Tween 80	DMA + PEG + glucose	rats	15	7.6	(62)
*Formulation 3*: mPEG-PCL^b^	30	–3.5	10	44.5% curcumin release after 100 h in PBS + 0.5% Tween 80 at pH = 7.4	NA	rats	50	7.7	(76)
*Formulation 4*: HA-curc-NC^d^	161	–25	3.3	15% release in PBS + 0.5% Tween at pH = 5.0. The release increased in the presence of HAase over 24 h. 40%, 60%, and 80% release within 24 h in PBS + 0.5% Tween containing 0.3 μM HAase at pH = 7.4 (blood), 6.5 (cancer site), and 5.0 (lysosome), respectively.	NA	SD rats	2	13.5	(47)
*Formulation 5*: mPEG–PLGA nanoparticles^e^	120	NA	NA	70% release within 27 h in PBS at pH = 5.8. Release reached 90% in 144 h	NA	SD rats	4	19	(43)
*Formulation 6*: mPEG-*b*-PHEMA-5HA micelles^f^	104	–19	17.8	90% and 80% release after 30 h in PBS at pH = 7.4 for noncross-linked and cross-linked micelles, respectively. Higher release (35%) for pH-sensitive cross-linked micelles in acidic environment (pH = 5.0) was observed compared to noncross-linked micelles (25%).	NA	SD rats	5	23	(46)
*Formulation 7*: Zein-PSBMA micelles^g^	155	–5.3	3.6	77% curcumin release in PBS, pH = 7.4, after 168 h.	saline with 1% Tween 20	BALB/c mice	2	37	(53)
*Formulation 8*: HA-Cur-liposomes^h^	210	–37	13.2	100% release after 100 h in PBS + 0.2% Tween 80 at pH = 7.4	DMSO	BALB/c mice	10	317	(40)
*Formulation 9*: PDLLA-G-based nanoparticles^i^	200	–0.8	NA	10% and 45% release after 168 h in PBS at pH = 7.4 and pH = 5.5, respectively, as a result of polymer degradation and higher stability of curcumin at acidic pH	37.5% PEG 400 v/v	SD rats	12	1,011	(48)

a Abbreviations: ZP, ζ potential; ID, injected dose; DMSO, dimethyl sulfoxide; DMA, dimethylacetamide; HA, hyaluronic acid; NA, not available; NC:C, curcumin nanoformulation:free curcumin; PBS, phosphate-buffered saline; PEG, polyethylene glycol; SD, Sprague–Dawley.

b Nanoformulation: monomethoxy poly(ethylene glycol)-poly(3-caprolactone).

c Nanoformulation: poly(ethylene glycol)-poly(lactic acid).

d Nanoformulation: hyaluronic acid-coated curcumin nanocrystals.

e Nanoformulation: (polyethylene glycol)-poly(lactic-*co*-glycolic acid).

f Nanoformulation: pH-responsive reversibly cross-linked micelles composed of poly(ethylene glycol)-*b*-poly(2-methacrylate ethyl 5-hexynoicate).

g Nanoformulation: zein-poly(sulfobetaine methacrylate).

h Nanoformulation: hyaluronic acid-modified liposomes.

i Nanoformulation: poly(d,l-lactic acid)-glycerol-based nanoparticles.

### 3.5 Physicochemical Characteristics of Curcumin Nanoformulations with Improved Pharmacokinetic Profiles

Studies on the well-performing formulations reported nanoformulated curcumin:free curcumin (NC:C) AUC ratios of 6, 7.6, 7.7, 13.5, 19, 23, and 37,^43,46,47,53,60,62,76^ where Sun et al.^40^ and Yoon et al.^48^ even obtained NC:C AUC ratios of 300 and 1,000, respectively (Figure 4C, green bars). The outstanding AUC values were also reflected in the *V*_d_ and CL (Figure 6, green bars). The type of nanoparticles and the physicochemical properties of these formulations are summarized in Table 2. The mean particle size ranged from 27 to 210 nm for the studies that reported an NC:C AUC ratio of >5,^40,43,46−48,53,60,62,76^ which is appropriate for prolonged circulation time.^110^ Moreover, the mean ζ potential of the particles was near-neutral (2.9 to −5.3 mV)^48,53,62,76^ or negative (<−10 mV).^40,46,47^

The reasons given for the improved AUC of curcumin nanoformulations compared to free curcumin in terms of physicochemical characteristics (Table 2) have mainly been attributed to improved chemical stability of curcumin by encapsulation and retention inside the nanoparticle. For instance, Liu et al.^46^ explained that higher micellar stability and better payload retention by the cross-linked micelles resulted in lower clearance of curcumin and thus a higher AUC. However, the authors did not report the *t*_1/2_ of these nanoparticles. Ji et al.^47^ argued that hyaluronic acid (HA) grafting onto curcumin nanocrystals using 1-ethyl-(3-dimethyl aminopropyl) carbodiimide improved particle stability and slowed down curcumin release (100% curcumin release within 1 h and 15% within 24 h in PBS + 0.5% Tween, pH = 5.0, for uncoated and HA-coated curcumin nanocrystals, respectively). Similarly, the long-circulating property of zein-poly(sulfobetaine methacrylate) micelles (zein-PSBMA) (half-life not reported) composed of zein (a protein extracted from corn) as the core and poly(sulfobetaine methacrylate) as the shell, in addition to the high retention of the loaded curcumin, resulted in a 37 times greater NC:C AUC ratio.^53^ Furthermore, sustained curcumin release from poly(d,l-lactic acid)-glycerol (PDLLA-G)-based nanoparticles was posited by Yoon et al.^48^ to account for the high NC:C AUC ratio. Generally, the improved PK of intravenously administered nanoformulations relies on the stability and low clearance rate of the nanoparticles, ensuring high cargo retention and prolonged circulation time, rather than on a sustained release mechanism. In this study^48^ with the highest reported NC:C AUC ratio of 1,000, the measured *C*_max_ of free curcumin was ∼0.1 μg/mL compared to 100 μg/mL for the nanoformulation at an equal injection dose of 12 mg/kg. Thus, it is likely that the high NC:C AUC ratio mainly resulted from the low AUC of free curcumin than from an extraordinary stability of the carrier system.

Of the studies that reported a high NC:C AUC ratio (Table 2), three studies performed *in vivo* imaging using a fluorescent tracer either encapsulated in or conjugated to the nanocarrier to assess circulation time. DIR-loaded cross-linked mPEG-*b*-PHEMA-5HA micelles (Table 2, *Formulation 6*) showed prolonged circulation time and higher tumor accumulation compared to the non-cross-linked micelles and free dye as controls.^46^ A drawback is that the *in vivo* imaging of DIR-loaded cross-linked mPEG-*b*-PHEMA-5HA micelles was performed in tumor-bearing mice, while the PK analysis was conducted in healthy rats. It is also remarkable that two HA-coated formulations^40,47^ were associated with a relatively high NC:C AUC ratio despite the fact that intravenously administered HA has a *t*_1/2_ of 2.5–4.5 min.^111^ Nonetheless, improved PK of nanoencapsulated curcumin has been ascribed to different types of HA-grafted nanoparticles. For instance, healthy mice received an intravenous injection of HA-coated liposomes loaded with DID (Table 2, *Formulation 8*), a lipophilic near-infrared fluorescent membrane dye, and were terminated after 12 h to select the optimal formulation in terms of HA molecular weight and grafting density based on organ uptake (especially the liver and spleen).^40^

Attachment of HA is not strictly necessary. HA-lacking Cy5.5-labeled zein-PSBMA micelles (Table 2, *Formulation 7*) showed more intense fluorescence than the control group (free Cy5.5) and the fluorescence signal was detectable 72 h after injection in mice. In contrast, the control group exhibited a significant decrease in the fluorescence after 6 h followed by signal disappearance after 48 h, indicating prolonged circulation of zein-PSBMA micelles.^53^ There are several stable nanoformulations with curcumin release of only ∼20% after 24 h that yielded NC:C AUC ratios in the range of 4.1–1,011.^40,44,47,48,58,60,62,71,76^

Unfortunately, none of the studies quantitatively reported the circulation kinetics of the curcumin nanocarrier and thus no firm conclusions can be drawn regarding the PK of curcumin versus its carrier system. Further research is therefore needed to understand particle stability in the circulation and in relation to the PK of nanoencapsulated curcumin. Above all, it is recommended to covalently attach the fluorescent dye to the nanocarrier because loaded dyes can be released or extracted from the carrier system and thwart data interpretation due to differential PK and disposition compared to the nano carrier.^112^

## 4 Improved AUC Does Not Necessarily Translate to Improved Therapeutic Efficacy

The main challenges that have been addressed above pertain to curcumin PK and center on curcumin retention in the circulation. It was concluded that certain nanoformulations are capable of improving the otherwise grim PK profile of curcumin following intravenous delivery. As blood vessels constitute the main conduit for a drug to reach a tumor, rapid exit of a drug from the bloodstream into tissues is detrimental to therapeutic efficacy. Given the poor retention of free curcumin in the circulation, intravenous administration of free curcumin is unlikely a viable approach to systemic therapy. Nanoformulations with stealth-like properties and effective tumor targeting are therefore the only potentially fruitful intervention strategy. The optimized PK associated with the nanoformulations under investigation does not by definition translate to (improved) PD efficacy. We therefore examined whether a correlation exists between PK and PD of free curcumin and curcumin nanoformulations using published studies that employed murine models of cancer. It was hypothesized that free curcumin would induce some TGI compared to vehicle control and that the antitumor activity would be exacerbated by nanoencapsulation. Moreover, it was expected that an increasing AUC, injected dose, and NC:C AUC ratio would (1) lead to higher antitumor activity (i.e., an increasing nominal difference in %TGI) and (2) widen the difference in %TGI between free versus nanoformulated curcumin.

The studies that met the inclusion criteria are summarized in Table 3. In a case-matched analysis, the animals that received curcumin nanoformulations exhibited more profound therapeutic effects than those that received free curcumin, which is in line with the increased AUC due to nanoencapsulation. Curcumin is cytostatic and cytotoxic to cancer cells^6^ but also interferes with other important features of tumor biology such as angiogenesis,^57,58,60,71,76^ which theoretically should lead to greater PD with improved PK. It must be noted that, in some studies,^46,47,57,58,60,61,76,77^ the PK parameters were reported in wild-type rats, while the therapeutic efficacy was determined in tumor-bearing immunocompromised mice.

**Table 3:** Summary of the Therapeutic Efficacy Studies of Intravenously Administered Curcumin Nanoformulations^a^

curcumin nanoformulation	mean size (nm)	mean ZP (mV)	animal model PK	animal model, xenograft PD	curcumin dosing schedule	monitoring time (d)	AUC ratio NC:C	%TGI (NC)	%TGI (C)	ref
curcumin-PBCA nanoparticles^b^	200	29	SD rats	athymic BALB/c nude mice, HepG2, s.c. flank	3 times per week for 28 days (curcumin dose unknown)	28	3.4	55	NA	(57)
mPEG-PLA micelles^c^	30	–0.3	SD rats	BALB/c mice, CT26 s.c. flank	50 mg/kg every 3 days for 15 days (total dose: 300 mg/kg)	25	5.0	80	60	(58)
mPEG-PCL-Phe(Boc) micelles^d^	23	NA	ICR mice	BALB/c nude mice, K562/ADR s.c. arm pit	40 mg/kg daily for 21 days (total dose: 840 mg/kg)	21	3.1	65	NA	(70)
mPEG-PCL micelles^e^	27	–0.8	C57BL/6 mice	C57BL/6 mice, LL/2 s.c. flank and pulmonary metastases i.v.	25 mg/kg every 2 days for 14 days (total dose: 200 mg/kg)	28	5.0	52	30	(71)
mPEG-PLA-PAE micelles^f^	pH = 7.4, 171;	pH = 7.4, 4;	BALB/c nude mice	BALB/c nude mice, MCF-7 s.c. flank	40 mg/kg on days 0, 2, 4, 6, and 8 (total dose: 200 mg/kg)	25	NA	65	NA	(41)
	pH = 5.5, 23	pH = 5.5, 25								
mPEG-PCL micelles^e^	27	NA	SD rats	BALB/c mice, C26 s.c.	25 mg/kg daily for 10 days (total dose: 250 mg/kg)	18	6.0	50	30	(60)
mPEG-b-PHEMA-5HA micelles^g^	104	–19	SD rats	BALB/c mice, 4T1 s.c.	20 mg/kg every 3 days for 21 days (total of 5 injections) (total dose: 100 mg/kg)	21	23	40	25	(46)
HA-curc-NC^h^	162	–25	SD rats	BALB/c mice, 4T1 s.c. flank breast	5 mg/kg every 2 days for 10 days (total dose: 30 mg/kg)	10	13.5	75	20	(47)
curcumin nanosuspension stabilized by mPEG-DSPE and SPC^i^	186	–19	SD rats	ICR mice, H22 s.c. armpit	10 mg/kg every other day (total dose: 40 mg/kg)	6	4.5	60	15	(61)
mPEG-PCL micelles^e^	30	–4	rats	BALB/c mice, CT26 s.c. flank	50 mg/kg every 2 days (total dose: 500 mg/kg)	18	7.7	20	16	(76)
HSA nanoparticles^j^	132	–21	rats	BALB/c mice, HT-29 s.c. dorsal flank	10 mg/kg every other day for 10 days (total dose: 60 mg/kg)	20	2.5	45	18	(77)

a The percentage of tumor growth inhibition (%TGI) is calculated as the difference in the ratio of tumor volume in the treatment group receiving curcumin nanoformulation compared to the untreated control group (%TGI (NC)) or the difference in the ratio of tumor volume of the treatment group receiving free curcumin versus the untreated control group (%TGI (C)) on the last day of tumor monitoring. Abbreviations: ZP, ζ potential; PK, pharmacokinetics; PD, pharmacodynamics; d, days; NC, curcumin nanoformulation; C, free curcumin; TGI, tumor growth inhibition; ref., reference; NA, not available; s.c., subcutaneous; i.v., intravenous. Cell lines: HepG2, human hepatocellular carcinoma; CT26, mouse fibroblasts from colon carcinoma; K562/ADR, human chronic myelogenous leukemia with selected resistance to doxorubicin; LL/2, mouse Lewis lung carcinoma; MCF-7, human mammary gland carcinoma; C26, mouse colon carcinoma; 4T1, mouse mammary gland carcinoma; H22, mouse hepatocellular carcinoma; HT-29, human colorectal adenocarcinoma.

b Nanoformulation: cationic poly(butyl) cyanoacrylate (PBCA) nanoparticles coated with chitosan.

c Nanoformulation: monomethoxy poly(ethylene glycol)poly(lactide).

d Nanoformulation: methoxy-poly(ethyleneglycol)-*block*-poly(ε-caprolactone) and *N*-(*tert*-butoxycarbonyl)-l-phenylalanine end-capped.

e Nanoformulation: methoxy poly(ethylene glycol)-*block*-poly(ε-caprolactone);

f Nanoformulation: pH-sensitive methoxy poly(ethylene glycol)-poly(lactide)-poly(β-amino ester).

g Nanoformulation: pH-responsive reversibly cross-linked micelles poly(ethylene glycol)-*b*-poly(2-methacrylate ethyl 5-hexynoicate).

h Nanoformulation: hyaluronic acid-modified curcumin nanocrystals;

i curcumin nanosuspension stabilized by mPEG2000-DSPE and soybean lecithin.

j Nanoformulation: human serum albumin nanoparticles.

**Figure 7:**
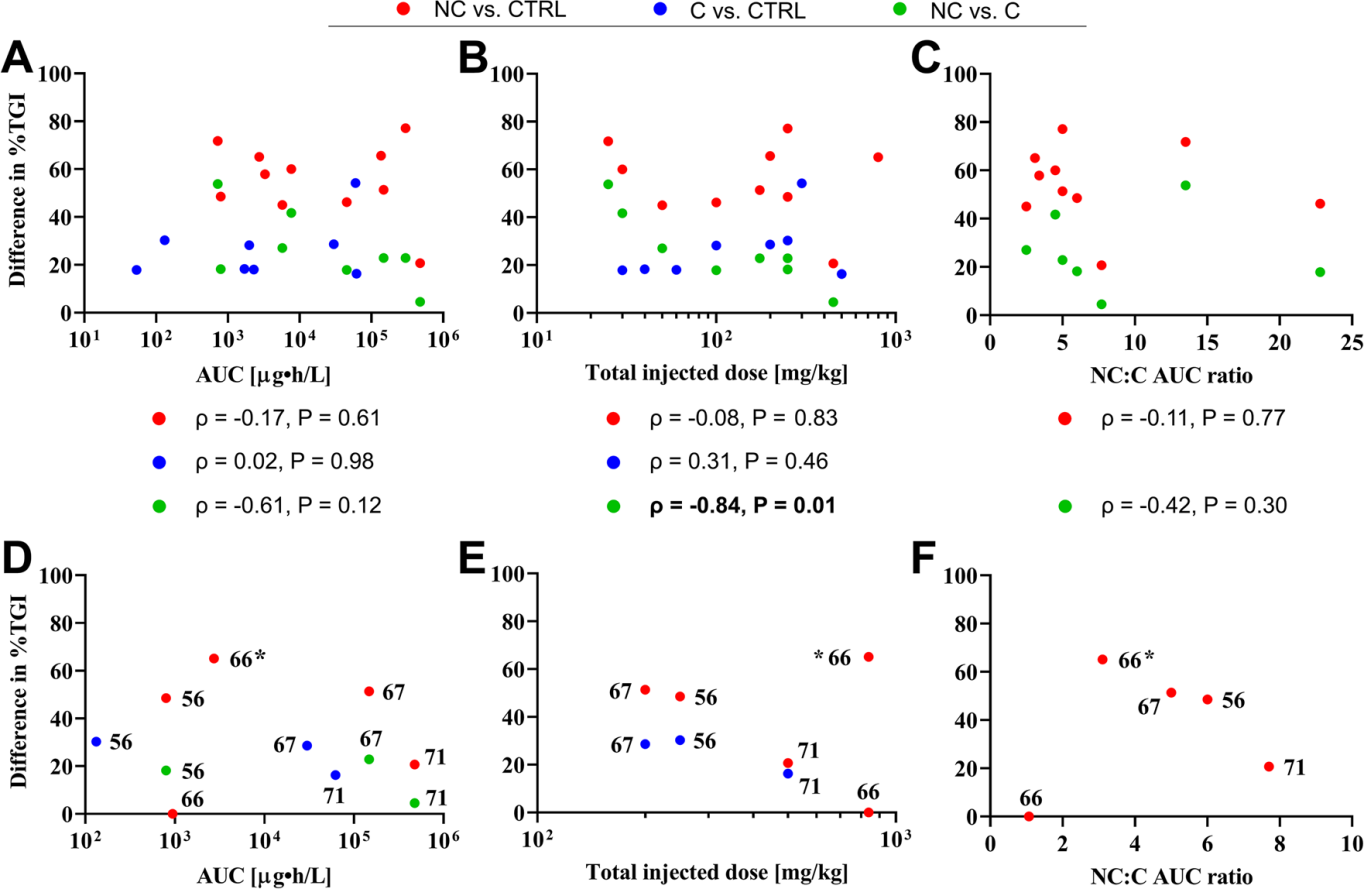
Correlation between therapeutic efficacy and AUC (A), total injected dose (B), and the nanoformulation curcumin:free curcumin (NC:C) ratio (C). The percentage of tumor growth inhibition (%TGI, *y*-axis) is provided as the nominal difference between %TGI of one group compared to another group (see legend), measured on the last day of tumor monitoring. The *x*-axis values in (A, B) pertain to NC (red circle, green circle) and C (blue circle) in the comparisons. The statistics (Spearman correlation coefficient, ρ, and *P*-value) of each comparison (legend) are presented below the panels. Panels (D–F) represent the same data sets as panels (A–C) but for curcumin-loaded mPEG-PCL micelles and free curcumin. The number next to each data point indicates the reference from which the data were collected.

To systematically assess the relationship between PK and PD in a clustered analysis, the nominal difference in %TGI achieved with free curcumin and curcumin nanoformulations (compared to respective controls) was plotted versus AUC, total injected dose, and the NC:C AUC ratio (Figure 7A-C) to derive Spearman’s correlation coefficient. These analyses give insight into the validity of the putative premises that (AUC ↑ → PD ↑) and (systemic concentration ↑ → PD ↑).

In terms of AUC, an increase in therapeutic efficacy (difference in %TGI) at higher AUCs was not observed when nanoformulated curcumin (NC) was compared to vehicle/solvent control, as evidenced by the lack of positive correlation (Figure 7A, red data points and statistics table below). The same applies to free curcumin (C), which further exhibited a generally lower AUC and nominal difference in %TGI compared to solvent control than the nanoformulated curcumin group (Figure 7A, blue data points). When NC was compared to C and plotted as a function of AUC (of NC), the therapeutic efficacy abated with improved PK of NC (Figure 7A, green data points) given the strong negative, albeit nonsignificant correlation. A clear explanation for this phenomenon is presently at large. It is possible that (1) there is a nonlinear relationship between circulation time and intratumoral NC delivery, (2) uptake of NC impedes cell death cascades compared to internalized C, (3) C accumulated in the tumor to a sufficient degree to induce cell death despite poor PK (during the distribution phase, especially in studies where micelle-forming surfactant molecules were used to solubilize free curcumin; see section 3.1), or (4) the NC did not extravasate from the blood vessels, hampering the delivery of cytotoxic cargo into the target cells. Other contributing factors may be the variety in the type of nanoformulations, tumor models, the discrepancy between PK/PD animal models, and/or different time frames that animals were monitored. Naturally, the eventual strength of the PK–PD correlation analysis relies on the translatability of curcumin PK in rats to the mouse situation, in which all the PD studies were conducted.

Comparable outcomes were observed when the difference in %TGI was plotted as a function of injected dose (Figure 7B). Administration of more NC or C did not yield more tumoricidal or cytostatic effects. Moreover, a strong and significant negative correlation was observed in the NC versus C comparison group when plotted as a function of injected dose (of NC). For this group, the dosages were matched. This finding is particularly striking in light of the fact that nanoencapsulation improves the measured *C*_max_ (section 3.1) and the AUC (section 3.2) while decreasing the *V*_d_ and CL for some formulations (section 3.4). An increasing injected dosage on top of those inherently advantageous properties was therefore expected to exacerbate the %TGI of NC and its divergence from the %TGI of C.

In the final set of analyses, the NC:C AUC ratio was calculated and plotted versus the difference in %TGI. In this approach the effect on PD is considered from a quantitative improvement in the PK of NC, which is then compared to vehicle/solvent control (Figure 7C, red data points) or to C (Figure 7C, green data points) in terms of anticancer efficacy. With this analysis, the potential %TGI-amplifying effects of a protracted AUC of C (due to micelle-forming solvent molecules) was corrected. Nevertheless, the nanoformulations with a relatively low NC:C AUC ratio performed equally well if not better than the more stable nanoformulations^57,58,61,70,71,77^ with a relatively high NC:C AUC ratio, dismissing the notion that a prolonged curcumin circulation time and thus higher AUC leads to better treatment outcome.

Lastly, the same set of analyses was performed for one type of polymer formulation to eliminate PK–PD differences owing to the use of different types of materials. Copolymers of methoxy poly(ethylene glycol)-*block*-poly(ε-caprolactone) (mPEG-PCL) are frequently used for the solubilization and delivery of hydrophobic drugs,^113^ including curcumin (Table 3). The eligible studies^60,70,71,76^ encompassed mPEG-PCL block copolymers with equal hydrophilic and hydrophobic molecular weights (2000 g/mol). The results are presented in Figure 7D–F.

The first notable observation from the data is that an injection dose range of only 300 mg/kg (200–500 mg/kg; Figure 7E) yielded an AUC spread of 3 orders of magnitude (Figure 7D) when assayed in the same animal species.^60,76^ Differences in the experimental and analytical techniques used might explain this discrepancy, which pleads for protocol standardization to enable interstudy comparisons. Second, a higher AUC (Figure 7D) or injected dose (Figure 7E) did not lead to improved therapeutic efficacy and generated contrasting data. Gong et al.^71^ and Gou et al.^60^ reported around 50% tumor reduction compared to the untreated group after administration of 25 mg/kg curcumin-loaded mPEG-PCL micelles every 2 days for 2 weeks in mice bearing LL/2 Lewis lung carcinoma and 25 mg/kg daily for 10 days in mice bearing C26 murine colon carcinoma tumor xenografts, respectively. Contrastingly, curcumin-loaded mPEG-PCL micelles did not exhibit tumor reduction compared to the control group (physiological saline) in mice bearing K567/ADR chronic myelogenous leukemia xenografts following a curcumin dosing schedule of 40 mg/kg every day for 3 weeks. This could be due to the similar PK of curcumin-loaded mPEG-PCL and free curcumin reported in this study (no difference in AUC was observed) and/or attributable to the multidrug resistance phenotype of K562/ADR cells. The *N*-(*tert*-butoxycarbonyl)-l-phenylalanine end-capped mPEG-PCL micelles with higher stability yielded a 65% tumor reduction compared to the untreated group.^70^ Third, despite uniformity in drug delivery systems, the AUC ratio of mPEG-PCL micelles to free curcumin was different among the studies. Gong et al.,^71^ Gou et al.^60^ and Hu et al.^76^ reported AUC ratios of 5.0, 6.0, and 7.7, respectively, whereas Gong et al. found an AUC ratio of 1.1.^70^ There was no evident positive correlation between PK and PD, altogether attesting to the main conclusion that, although nanoformulations improve curcumin PK (AUC), this does not as a rule result in greater oncotherapeutic efficacy.

## 5 Preliminary Translational and Clinical Outcomes of Intravenous Curcumin Nanoformulations

Presently there are only two intravenous curcumin nanoformulations (Lipocurc and CUC-01) registered in the clinicaltrials.gov database for cancer treatment. To date, the efficacy and safety of CUC-01, a curcuminoid formulation in polyoxyl castor oil (Kolliphor ELP) as a nonionic solubilizer, was evaluated in patients with metastatic breast cancer in combination with paclitaxel following intravenous administration of both. As explained earlier, using such surfactants as solubilizers results in micellar nanoformulations. The authors reported that the combination therapy was superior to paclitaxel plus placebo without major safety concerns.^114^

The PK and biodistribution profile of Lipocurc, a liposomal curcumin formulation, have been extensively investigated in dogs and humans in several studies. The impact of the duration of intravenous infusion of Lipocurc on curcumin metabolism and tissue distribution was assessed in dogs. The tissue levels of curcumin and its metabolite tetrahydrocurcumin in the lungs, spleen, and liver were substantially higher after the 8-h regimen compared to the 2-h regimen. Also, the longer infusion time resulted in a higher tissue partition coefficient for curcumin and tetrahydrocurcumin. The ratio of the metabolite to curcumin was lower during longer infusion regimens and different in a tissue-specific manner. The authors argued that the extended infusion might facilitate the distribution of curcumin into tissues by a transporter-dependent mechanism and that higher tissue concentrations of curcumin might inhibit or saturate a putative reductase enzyme that converts curcumin to its metabolite.^115^ However, later the authors suggested another mechanism to substantiate these observations by performing complementary *in vitro* experiments that are discussed below.^81,116,117^

The pharmacokinetic profile, safety, and tolerability of Lipocurc were studied in a phase I dose escalation trial following a single bolus intravenous injection in the range of 10–400 mg/m^2^. The plasma concentration of curcumin and tetrahydrocurcumin increased in a dose-dependent manner. Shortly after discontinuation of the infusion (6–60 min), the curcumin concentration in plasma fell below the detection limit. Intravenous dosing was safe and above 120 mg/m^2^ a transient change in the morphology of red blood cells (RBCs) was noticed. Therefore, short-term infusion of Lipocurc was deemed safe up to 120 mg/m^2^, and higher doses represented dose-limiting toxicity in RBCs.^116^ In subsequent experiments the authors confirmed *in vitro* that curcumin and liposomal curcumin caused morphological changes in RBCs in a dose-dependent manner.^117^

In the wake of these observations, the cellular distribution and metabolism of curcumin (formulated as Lipocure) were investigated *in vitro* in RBCs and peripheral blood mononuclear cells (PBMCs). It was demonstrated that curcumin rapidly distributed into RBCs and PBMCs. The authors assumed that Lipocurc adsorbs onto the cell membrane of RBC/PBMCs, after which curcumin diffuses across the cell membrane into the intracellular compartment. Blood-based metabolism, in particular in RBCs, was observed as 92% and 68% of curcumin disappeared from the medium as soon as 15 min after the addition of canine and human RBCs, respectively. Thus, incorporation of curcumin and subsequent metabolism into tetrahydrocurcumin and possibly other metabolites occurs in human and canine RBCs. Although the type of enzyme(s) responsible for the metabolism is not yet clear, the authors proposed an enzyme comparable to dihydrocurcumin reductase in gut microorganisms or cytochrome b5 reductase that is present in mammalian cells, including RBCs.^118^

The findings are relevant for the PK profile of curcumin. Together with organ-based metabolism and possibly chemical instability, the data explain why steady-state levels of curcumin were not achieved after infusion and provide a rationale for the short plasma half-lives. The authors hypothesized that RBCs might serve as a vehicle to distribute curcumin to tissues due to the cell’s fractional abundance in blood. This, according to the authors, may explain the higher curcumin concentration in tissues after eight-hour infusion compared to two-hour infusion. When reported in terms of per cell basis, curcumin was retrieved at a higher concentration in PBMCs compared to RBCs, which can be of potential therapeutic utility in the treatment of tumors with lymphocytic origin.^81^ Following up on this observation, the authors demonstrated higher curcumin distribution into PBMCs in chronic lymphocytic leukemia patient-derived PBMCs compared to healthy donors^119^ and higher uptake in multiple myeloma cell lines,^120^ underpinning a potential therapeutic benefit in the treatment of hematological cancers. Therefore, the role of RBCs in the PK and PD of curcumin should be evaluated carefully. Apart from being an intrinsic carrier, accumulation and subsequent metabolism of curcumin may yield less active metabolites that are ready to be cleared.

## 6 Conclusions and Outlook

Curcumin is a compound with PD potency but PK frailty, characterized by rapid removal from plasma during the distribution phase and the absence of steady-state plasma levels after intravenous administration. Consequently, curcumin’s PD potency does not come to fruition *in vivo*. Our systematic analysis of animal studies revealed that curcumin loading into nanocarriers is beneficial for the compound’s PK profile in that nanoencapsulation improved the measured *C*_max_ and AUC while reducing the *V*_d_ and CL compared to the free form. However, these effects were not ubiquitous and depended on the nanoformulation type. Also, certain curcumin nanoformulations were associated with a greater %TGI compared to free curcumin in different tumor models, which is in line with the improved PK profile. However, clustered analysis revealed that there is no positive correlation between AUC as well as injected dose and antitumor efficacy. In addition, almost all studies neglected to investigate the nanocarrier’s PK profile and ability to deliver cargo into the target cells. Those few papers that followed up on the circulation kinetics of the nanocarrier did not report the data quantitatively. Therefore, at this point, it is not possible to firmly conclude whether stable nanoformulations with high curcumin retention are required for better therapeutic efficacy than nanoformulations that are merely solubilizers. It is therefore recommended that future investigations on curcumin nanoformulations as oncotherapeutics go beyond the classical PK parameters and focus mainly on intratumoral curcumin levels as a function of time after administration and corollary effects on tumor volume or mass. Based on our analysis, the classical PK parameters are inadequate and cannot be employed as barometers for therapeutic efficacy, rendering studies that focus solely on the PK of curcumin nanoformulations equally inadequate in broader context.

The rapid distribution of curcumin into blood cells leads to compartmentalization that could skew conclusions regarding curcumin bioavailability (in case of oral formulations) and circulation time. Whole blood-based assays should be developed and used to quantitate curcumin levels in blood. It is also possible that curcumin exploits blood cells as temporary carriers and eventually redistributes from the cellular compartment to plasma or is trafficked into the tumor microenvironment via a cellular carrier. Acquiring a more thorough understanding of these phenomena will spawn higher-resolution insight into curcumin’s PK–PD relationship in terms of solid malignancies. Further room for improvement lies in optimizing administration regimens, where injection over a longer period of time has shown promise in dogs in regard to more profound tissue accumulation.^115^ For hematological malignancies, on the other hand, the blood cell-occupying behavior of curcumin may de facto be conducive to treatment efficacy.

## Supporting Information Available

Details regarding pharmacokinetics principles and calculation methods, tables containing raw data of PK parameters extracted from literature, and comparison between calculated and measured AUC values *in vivo* for free curcumin and curcumin nanoformulations (PDF)

## Abbreviations

AUC: area under the curve
PK: pharmacokinetics
PD: pharmacodynamic
TGI: tumor growth inhibition
S: Supporting Information
*C*_max_: maximum concentration
*t*_1/2_: elimination half-life
CL: clearance
*V*_d_: distribution volume
CN: curcumin nanoformulation
C: free curcumin
PBS: phosphate buffered saline, RBC, red blood cell
HA: hyaluronic acid
ZP: ζ potential
ID: injected dose
DMSO: dimethyl sulfoxide
PEG: polyethylene glycol
d: days
NA: not available
SD: Sprague–Dawley
s.c.: subcutaneous
i.v.: intravenous
CTRL: control
PBMCs: peripheral blood mononuclear cells
HA-curc-NC: hyaluronic acid-coated curcumin nanocrystals
mPEG-PLGA: (polyethylene glycol)-poly(lactic-*co*-glycolic acid)
mPEG-b-PHEMA-5HA micelles: pH-responsive reversibly cross-linked micelles poly(ethylene glycol)-*b*-poly(2-methacrylate ethyl 5-hexynoicate)
zein-PSBMA micelles: zein-poly(sulfobetaine methacrylate)
HA-Cur-LPs: hyaluronic acid modified liposomes
(PDLLA-G)-based nanoparticles: poly(d,l-lactic acid)-glycerol-based nanoparticles
curcumin-PBCA nanoparticles: cationic poly(butyl) cyanoacrylate nanoparticles coated with chitosan
mPEG-PLA: monomethoxy poly(ethylene glycol)poly(lactide)
mPEG-PCL-Phe(Boc) micelles: methoxy-poly(ethyleneglycol)-*block*-poly(ε-caprolactone) and *N*-(*tert*-butoxycarbonyl)-l-phenylalanine end-capped
mPEG-PCL: methoxy poly(ethylene glycol)-*block*-poly(ε-caprolactone)
mPEG-PLA-PAE: pH-sensitive methoxy poly(ethylene glycol)-poly(lactide)-poly(β-amino ester)
mPEG-*b*-PHEMA-5HA: pH-responsive reversibly cross-linked micelles poly(ethylene glycol)-*b*-poly(2-methacrylate ethyl 5-hexynoicate)
HA-curc-NC: hyaluronic acid-modified curcumin nanocrystals
mPEG-DSPE: monomethoxy poly(ethylene glycol)-distearoyl phosphatidylcholine
SPC: soybean lecithin
HSA: human serum albumin
