# Supplementary material for: Utility of Intravenous Curcumin Nanodelivery Systems for Improving In Vivo Pharmacokinetics and Anticancer Pharmacodynamics
Source: Mol Pharm. 2022 Aug 16;19(9):3057–74. doi: 10.1021/acs.molpharmaceut.2c00455 (PMC9450039; doi:10.1021/acs.molpharmaceut.2c00455)
Supplement: Supplementary file 1 — mp2c00455_si_001.pdf [file mp2c00455_si_001.pdf]

SUPPLEMENTAL INFORMATION

Utility of Intravenous Curcumin Nanodelivery Systems for Improving *In Vivo* Pharmacokinetics and Anticancer Pharmacodynamics

Mahsa Bagheri[a], Cornelus F. van Nostrum[a], Robbert Jan Kok[a], Gert Storm[a], Wim E. Hennink[a], Michal Heger[a,b]*

[a] *Department of Pharmaceutics, Utrecht Institute for Pharmaceutical Sciences, Utrecht University, the Netherlands*
[b] *Department of Pharmaceutics, Jiaxing Key Laboratory for Photonanomedicine and Experimental Therapeutics, College of Medicine, Jiaxing University, Jiaxing 314001, Zhejiang, P. R. China*

S2. Data curation and analysis

Different PK models are used to describe how an organism handles the transformations and fate of a drug following exposure (e.g., after enteral or parenteral administration). The models can be classified into the non-compartmental model and compartmental models. Compartmental models can in turn be subclassified into single-compartmental-, two-compartmental-, and three- or multi-compartmental models.

S2.1. Non-compartmental model

The non-compartmental model considers the organism as a singular homogenous compartment and assumes that a compound's plasma concentration reflects the compound's concentration in other tissues. The compound's elimination is inversely proportional to its concentration in the organism. Empirical data such as plasma concentration over time serve as input variables to calculate PK parameters such as AUC, CL, C_{max}, T_{max}, and $t_{1/2}$.

S2.2.1. Single-compartment model

The single-compartment model (Figure S1) considers a central compartment to be comprised of all organs and tissues. The compound enters the central compartment after administration and then exits the central compartment, whereby recirculation of the compound does not occur.

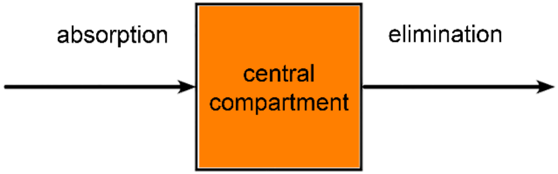

Figure S1. Schematic illustration of the single-compartment model. Note that the absorption phase pertains only to orally administered drugs and not intravenously delivered drugs.

S2.2.2. Two-compartment model

The two-compartment model (Figure S2) is predicated on the proposition that the organs and tissues are divided into a central compartment and a peripheral compartment. The plasma concentration of the compound decays through multiple exponential phases.

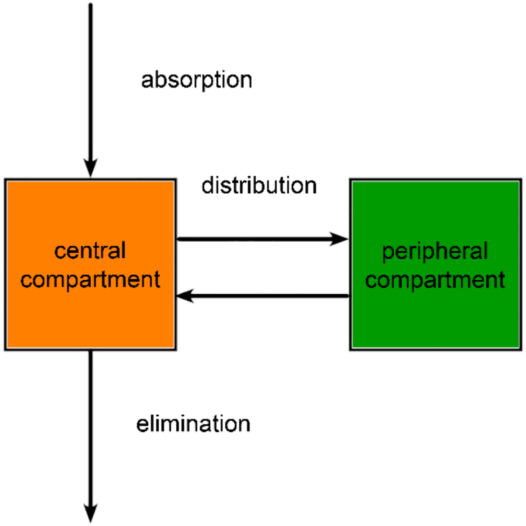

Figure S2. Schematic illustration of the two-compartment model. Note that the absorption phase pertains only to orally administered drugs and not intravenously delivered drugs.

S2.2.3. Multi-compartment model

In the multi-compartment model (Figure S3) the central compartment constitutes the circulation; the highly perfused compartment encompasses highly perfused organs and tissues, and the scarcely perfused compartment represents the poorly perfused organs and tissues.

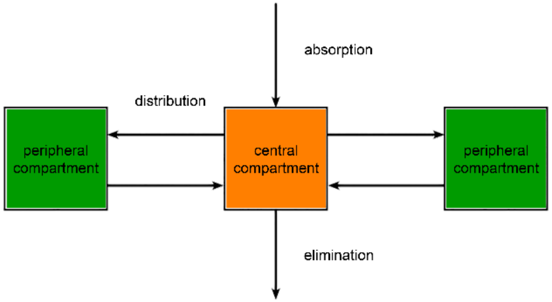

Figure S3. Schematic illustration of the multi-compartment model. Note that the absorption phase pertains only to orally administered drugs and not intravenously delivered drugs.

The not-reported clearance and volume of distribution are calculated as follows:

The reported $t_{1/2}$ or $t_{1/2\beta}$ was used in the equations
Clearance (CL) = dose/AUC, CL = $k \cdot V_d$, whereby $k = 0.693/t_{1/2}$
CL unit: volume/time/kg
Dose (D) unit: amount

Elimination half-life ($t_{1/2}$) unit: time
Area under the plasma concentration-time curve (AUC) unit: amount•time/volume
Elimination rate constant (k) unit: $time^{-1}$
Apparent volume of distribution (V_d) unit: volume/kg

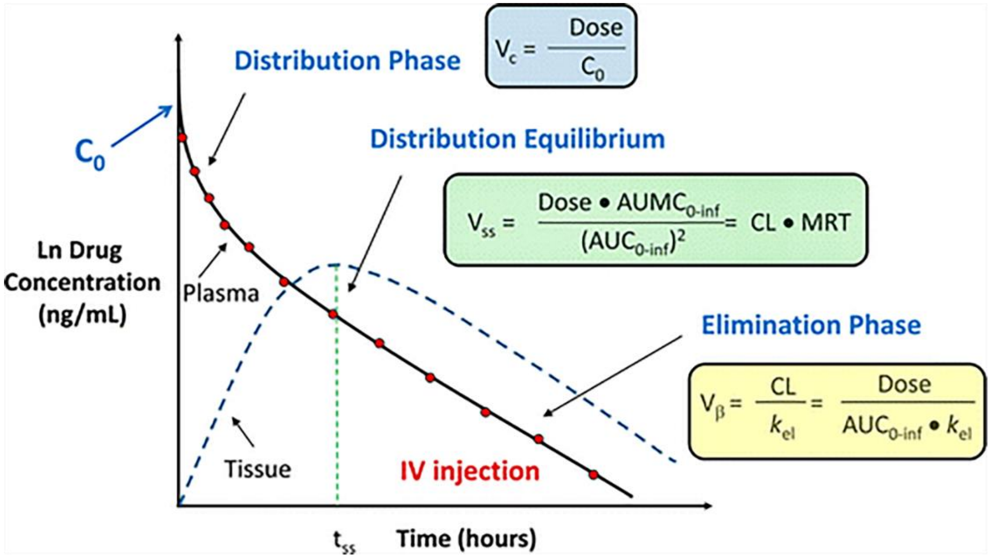

Figure S4. Definition of the different volumes of distribution of drugs after IV administration. Reprinted with permission from reference [1]. Copyright 2015, American Chemical Society. Abbreviations: AUC, area under the plasma concentration versus time curve; AUMC, area under the first-moment curve; CL, clearance; k_{el}, elimination rate constant; MRT, mean residence time; V_β, volume of distribution at pseudodistribution equilibrium; V_{ss}, volume of distribution at steady state; V_c, volume of the central compartment, t_{ss}, time to steady-state.

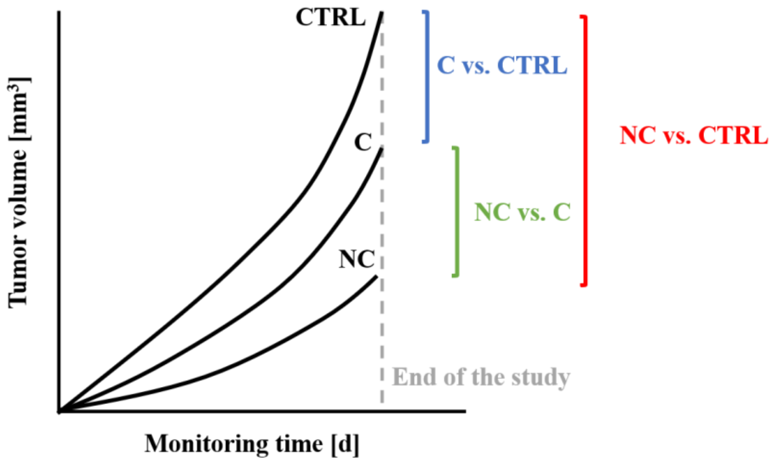

Figure S5. Schematic explanation of the approach to therapeutic efficacy analysis. The percentage of tumor growth inhibition (%TGI) was calculated based on the nominal difference between different treatment groups on the last day of tumor monitoring. The colors match the legends used in Figure 7. The control group comprises the administration of buffer or vehicle only, C is free curcumin, and NC represents treatment with a curcumin nanoformulation.

S3. Nanoformulations improve multiple curcumin pharmacokinetics parameters compared to non-formulated, free curcumin

S3.1. Pharmacokinetics of intravenously administered free curcumin

The free curcumin controls used in the *in vivo* studies were prepared using a solvent or solvent mixture comprising mainly DMSO [2-6], DMA + PEG400 + isotonic dextrose or glucose [7-16], or solubilizers such as Kolliphor EL (the new name for Cremophor EL) [17-19], Kolliphor HS15 [20], Tween 20 [21] or Tween 80 [22]. The type of vehicle can impact the PK profile which has already been demonstrated for Kolliphor as a vehicle of hydrophobic drugs. The PK of paclitaxel formulated in Kolliphor EL was unpredictable and non-linear in plasma, probably due to the entrapment of paclitaxel in the Kolliphor EL micelles, leading to lower clearance and prolonged exposure [23-26].

Also, PK calculations were based on different sampling time periods. Most of the studies perform sampling during 12 h [2, 7, 10, 13, 14, 16, 27-30], 24 h [5, 6, 8, 9, 11, 15, 17-22, 31-36], 48 h [3, 37], or 72 h [4]. One study reported PK parameters ($AUC_{0\text{-inf}}$ and $t_{1/2}$) based on data acquired during the first hour after administration [12]; This study was therefore excluded from the analysis. Differences in sampling time could influence the reliability of the PK values due to the poor characterization of the initial or terminal elimination rate [38]. To explain, PK analysis should include a sufficient number of sample acquisition points during the first hour after intravenous administration to properly cover the distribution phase. Similarly, sampling should continue until curcumin is no longer detected in the plasma to fully account for the elimination phase. The same issues were underscored in a recent review of clinical studies in which different sampling times after oral curcumin administration had been used [39].

S3.3. Curcumin AUC is improved by nano-encapsulation

Table S1. Summary of PK parameters for intravenously administered free curcumin in mice (n = 7).

Solvent system	Dose [mg/kg]	$t_{1/2}$ [h]	C_{max} [μg/mL]	AUC [μg•h/L]	Normalized AUC [μg•h/L]/[mg/kg]	k [h^{-1}]	CL [L/h/kg]	V_d [L/kg]	Ref.
DMSO	12	0.2	0.003	878	73	3.7	13.7	3.8	[2]
N.A.	25	1.5	33.9	29,894	1,196	0.45	0.84	1.8*	[31]
Mixture	10	$t_{1/2\alpha}$ = 0.02; $t_{1/2\beta}$ = 0.3	3.7	474	47	2.7	21.1	7.7*	[7]
Mixture	10	$t_{1/2\alpha}$ = 0.02; $t_{1/2\beta}$ = 0.9	3.7	361	36	0.8	29	35.3*	[8]
DMSO	10	N.A.	~0.01	407	41	N.A.	5.8	N.A.	[4]
Mixture	2	$t_{1/2\alpha}$ = 0.02; $t_{1/2\beta}$ = 1.5	0.15	6,708	3,354	0.5	0.3	0.6*	[15]
Saline + 1% Tween 20	2	0.3	13	8,150	4,075	2.4	0.3*	0.1*	[21]

The calculated clearance (CL) and volume of distribution (V_d) values using the above formula are indicated by an asterisk. The solvent mixture that was used to solubilize free curcumin contained 15% DMA + 45% PEG + 40% dextrose or glucose.

Abbreviations: $t_{1/2}$, elimination half-life; C_{max}, maximum concentration; k, elimination rate constant; AUC, area under the plasma concentration versus time curve; CL, clearance; V_d, volume of distribution; Ref., reference; N.A., not available; DMA, dimethylformamide; DMSO, dimethyl sulfoxide; PEG, polyethylene glycol

Table S2. Summary of PK parameters of intravenously administered curcumin nanoformulations in mice (n = 9).

Formulation	Size [nm]	ZP [mV]	Dose [mg/kg]	$t_{1/2}$ [h]	Cmax [μg/mL]	AUC [μg•h/L]	Normalized AUC [μg•h/L]/[mg/kg]	k [h^{-1}]	CL [L/h/kg]	V_d [L/kg]	Ref.
mPEG-PCL-Phe(Boc) micelles[a]	23	N.A.	12	1.2	0.01	2,730	228	0.6	4.4	7.8	[2]
mPEG-PCL micelles[b]	37	-0.8	25	2.2	83.0	148,079	5,923	0.3	0.2	0.5*	[31]
mPEG-PLA-PAE micelles[c]	171	4	40	$t_{1/2a}$ = 0.14; $t_{1/2β}$ = 1.03	~170	136,228	3,405	0.7	5.9	7.2	[32]
PLGA-PEG-PLGA micelles[d]	26	-0.7	10	$t_{1/2α}$ = 0.057; $t_{1/2β}$ = 1.15	2.2	622	62	0.6	16.1	26.8*	[7]
PBCN nanoparticles[e]	152	0	5	$t_{1/2α}$ = 10.8; $t_{1/2β}$ = 33.1	1.3	758	152	0.02	7.5	315*	[8]
HA-Curc-LPs[f]	210	-36.8	10	N.A.	~31.6	128,998	12,900	N.A.	0.1	N.A.	[4]
PCL-PDEA-PSBMA micelles[g]	143	40	2	$t_{1/2a}$ = 0.2; $t_{1/2β}$ = 6.5	0.19	8,964	4,482	0.11	0.2	2.1*	[15]
Zein-PSBMA-based micelles[h]	155	-5.3	2	6.4	27	299,410	149,705	0.11	0.006*	0.06*	[21]
mPEG-*b*-p(HPMA-Bz)[i]	59	N.A.	50	$t_{1/2a}$ = 0.11; $t_{1/2β}$ = 2.5	N.A.	319,000	6,380	0.28	0.16	0.34	[40]

The calculated clearance (CL) and volume of distribution (V_d) values using the above formula are indicated by an asterisk.
Abbreviations: $t_{1/2}$, elimination half-life; C_{max}, maximum concentration; k, elimination rate constant; AUC, area under the plasma concentration versus time curve; CL, clearance; V_d, volume of distribution; ZP, zeta potential; Ref., reference; N.A., not available
Notes nanoformulations: [a] N-(tert-butoxycarbonyl)-L-phenylalanine end-capped methoxy-poly(ethylene glycol)-*b*-poly(ε-caprolactone); [b] monomethyl poly(ethylene glycol)-poly(ε-caprolactone); [c] pH-sensitive methoxy poly(ethylene glycol)-poly(lactide)-poly(β-amino ester); [d] poly(D,L-lactide-co-glycolide)-*b*-poly(ethylene glycol)-*b*-poly(D,L-lactide-co-glycolide); [e] polybutylcyanoacrylate nanoparticles coated with polysorbate 80; [f] curcumin liposomes modified with hyaluronic acid; [g] pH-sensitive poly(ε-caprolactone)-*b*-poly(diethylaminoethyl methacrylate)-*b*-poly(sulfobetaine methacrylate); [h] zein-poly(sulfobetaine methacrylate); [I] poly(ethylene glycol)-b-poly(N-2-benzoyloxypropyl methacrylamide)

Table S3. Summary of PK parameters of intravenously administered free curcumin in rats (n = 24).

Solvent system	Dose [mg/kg]	$t_{1/2}$ [h]	C_{max} [μg/mL]	AUC [μg•h/L]	Normalized AUC [μg•h/L]/[mg/kg]	K [h^{-1}]	CL [L/h/kg]	V_d [L/kg]	Ref.
N.A.	10	$t_{1/2\beta}$ = 0.4	N.A.	1,922	192	1.9	0.005	0.002	[41]
Cremophor	50	1.5	67.7	60,325	1,206	0.5	0.8*	1.8*	[17]
Cremophor	100	0.33	306	132	1	2.1	755*	355*	[18]
N.A.	5	$t_{1/2\beta}$ = 0.44	~0.25	1,990	398	1.6	2.5	15.8	[27]
N.A.	2	11.13	0.02	54	27	0.06	77#	1.2	[34]
DMSO:Tween 80 (1:1 *v/v*)	10	1.81	3.7	1,705	171	0.4	3.8	15.3*	[22]

Solvent system	Dose [mg/kg]	$t_{1/2}$ [h]	C_{max} [µg/mL]	AUC [µg•h/L]	Normalized AUC [µg•h/L]/[mg/kg]	K [h^{-1}]	CL [L/h/kg]	V_d [L/kg]	Ref.
N.A.	50	0.81	61.7	62,280	1,246	0.9	0.8*	0.9*	[35]
Cremophor	10	0.08	1.8	2,298	230	8.7	16.7	0.5*	[19]
N.A.	4	0.77	0.01	3	1	0.9	1238*	1376*	[37]
N.A.	6	3.6	~2.6	3,956	659	0.2	11.2	7.9*	[33]
DMSO	2	2.41	0.12	110	55	0.3	28.5	62.8*	[5]
Kolliphor HS15	10	1.15	13.1	18,770	1,877	0.6	$0.13^{\$}$	0.9*	[20]
Mixture	10	0.57	~10	1,670,000	167,000	1.2	5.5	2.1	[9]
PEG400	10	N.A.	~0.1	72	7	N.A.	17.0	N.A.	[30]
Mixture	15	0.09	19.8	109	7	7.7	137*	17.8*	[10]
37.5% (*v/v*) PEG400	12	0.11	~0.1	30	3	6.3	409	62	[28]
Mixture	10	$t_{1/2\alpha}$ = 0.133	3.7	8,688	869	N.A.	1.2	N.A.	[11]

Solvent system	Dose [mg/kg]	$t_{1/2}$ [h]	C_{max} [μg/mL]	AUC [μg•h/L]	Normalized AUC [μg•h/L]/[mg/kg]	K [h^{-1}]	CL [L/h/kg]	V_d [L/kg]	Ref.
N.A.	15	0.31	~3	835	56	2.2	18.0	8.1	[29]
Mixture	2	0.22	N.A.	112	56	3.21	18.1	5.7	[12]
Mixture	15	0.2	~4	967	65	3.5	15.5	4.4	[13]
Mixture	15	0.14	~2.5	668,415	44,561	5.1	0.022	0.004	[14]
DMSO	10	$t_{1/2\alpha} =$ 0.068<br>$t_{1/2\beta} =$ 0.56	5.1	1,250	125	1.24	4.1	0.8	[6]
N.A.	4	$t_{1/2\beta}$ = 1.1	0.002	1,697	424	0.7	2.4	3.6*	[36]
Mixture	15	0.2	~4.5	967	65	3.5	15.5	4.4	[16]

The calculated clearance (CL) and volume of distribution (V_d) values using the above formula are indicated by an asterisk. The solvent mixture that was used to solubilized free curcumin contained 15% DMA + 45% PEG + 40% dextrose or glucose.

Reported as 0.077 mL/h/ng

$ Reported as 0.13 (mg)/(μg/mL)/h)

Abbreviations: $t_{1/2}$, elimination half-life; C_{max}, maximum concentration; k, elimination rate constant; AUC, area under the plasma concentration versus time curve; CL, clearance; V_d, volume of distribution; Ref., reference; N.A., not available; DMA, dimethylformamide; DMSO, dimethyl sulfoxide; PEG, polyethylene glycol

Table S4. Summary of PK parameters of intravenously administered curcumin nanoformulations in rats (n = 23).

Formulation	Size [nm]	ZP [mV]	Dose [mg/kg]	$t_{1/2}$ [h]	C_{max} [μg/mL]	AUC [μg•h/L]	Normalized AUC [μg•h/L]/[mg/kg]	k [h^{-1}]	CL [L/h/kg]	V_d [L/kg]	Ref.
Curcumin-PBCA nanoparticles[a]	200	29.1	5	$t_{1/2\beta}$ = 18.7	N.A.	3,302	660	0.04	0.002	0.103	[41]
mPEG-PLA micelles[b]	30	-0.3	50	2.4	166	300,125	6,003	0.3	0.2*	0.6*	[17]
mPEG-PCL micelles[c]	27	N.A.	100	0.6	431	798	8	1.2	125*	103*	[18]
mPEG-*b*-PHEMA-5HA[d]	104	-19	5	$t_{1/2\beta}$ = 6.2	~1.1	45,340	9,068	0.11	0.11	0.3	[27]
HA-Curc-NC[e]	161	-25	2	53.1	0.08	724	362	0.01	6.0[#]	0.4	[34]
Curcumin nanosuspension stabilized by mPEG-DSPE and SPC[f]	186	-19	10	65	1.5	7,672	767	0.01	0.21	122*	[22]
mPEG-PCL[c]	30	-3.6	50	1.4	147	478,600	9,572	0.49	0.1*	0.2*	[35]
HSA nanoparticles[g]	165	-27.3	10	0.4	1	5,761	576	1.9	3.2	0.9*	[19]
Polymeric nanoparticles composed of NIPAAM, VP, and AA[h]	92	-20.1	10	3.2	25.5	151,135	15,113	0.22	0.07	0.3	[3]

Formulation	Size [nm]	ZP [mV]	Dose [mg/kg]	$t_{1/2}$ [h]	C_{max} [μg/mL]	AUC [μg•h/L]	Normalized AUC [μg•h/L]/[mg/kg]	k [h^{-1}]	CL [L/h/kg]	V_d [L/kg]	Ref.
mPEG-PLGA nanoparticles[i]	120	N.A.	4	9.1	0.02	59	15	0.08	67*	882*	[37]
Liposomes	130	-6.3	6	6.1	~2.1	6,123	1,021	0.11	6.2	8.6*	[33]
Tri-CL-mPEG nanoparticles[j]	116	-12.2	2	5.0	1.45	456	228	0.1	4.1	31.6*	[5]
Lipid nanocapsules	55	-11	10	3.3	70.1	72,980	7,298	0.2	0.04$	0.6*	[20]
MePEO-*b*-PCL micelles[k]	78	N.A.	5	61	~0.7	3,160,000	632,000	0.01	1.8	147	[9]
Nanoparticles based on CSH/HA/PEG[l]	246	-27	10	N.A.	~0.3	287	29	N.A.	4.5	N.A.	[30]
mPEG-PLA micelles[b]	70	3	15	0.3	20.7	835	56	2.4	18.0*	7.5*	[10]
(PDLLA-G)-based nanoparticles[m]	200	-0.8	12	1.43	~100	30,168	2,514	0.48	0.4	0.1	[28]
PCL-PDEASB micelles[n]	103	56.6	10	$t_{1/2\alpha}$ = 0.2	11.6	17,376	1,737	N.A.	0.6	N.A.	[11]
mPEG–PVL nanoparticles[o]	132	-2.1	15	5.1	~10	3,006	200	0.1	5.0	36.8	[29]
SLN[p]	153	-21.4	2	0.3	N.A.	139	70	2.1	14.5	6.9	[12]

Formulation	Size [nm]	ZP [mV]	Dose [mg/kg]	$t_{1/2}$ [h]	C_{max} [μg/mL]	AUC [μg•h/L]	Normalized AUC [μg•h/L]/[mg/kg]	k [h^{-1}]	CL [L/h/kg]	V_d [L/kg]	Ref.
Linear-dendrimer mPEG-PCL micelles[q]	108	-9.3	15	1.3	~15	4,464	298	0.54	3.4	6.3	[13]
PCL-PEG-PCL triblock nanoparticles[r]	62	-4.3	15	27.6	~2.5	2,772,523	184,835	0.03	0.005	0.21	[14]
mPEG-chitosan-Ketal micelles	50	N.A.	10	$t_{1/2a}$ = 0.21; $t_{1/2β}$ = 3.7	2.3	2,530	253	0.2	2.0	2.0	[6]
Lipid-polymer-lecithin hybrid nanoparticles	86	-26.9	4	5.9	0.002	3,416	854	0.12	1.2	10.0*	[36]
Linolenic acid-modified PEG-PCL micelles	21	-2.6	15	3.2	~9	2,662	177	0.22	5.6	25.6	[16]

The calculated clearance (CL) and volume of distribution (V_d) values using the above formula are indicated by an asterisk.

[#] Reported as 0.006 mL/h/ng

[$] Reported as 0.04 (mg)/(μg/mL)/h)

Abbreviations: $t_{1/2}$, elimination half-life; C_{max}, maximum concentration; k, elimination rate constant; AUC, area under the plasma concentration versus time curve; CL, clearance; V_d, volume of distribution; ZP, zeta potential; Ref., reference; N.A., not available

Notes nanoformulations: [a] cationic poly(butyl) cyanoacrylate (PBCA) nanoparticles coated with chitosan; [b] monomethoxy poly(ethylene glycol)poly(lactide) copolymer; [c] monomethoxy poly(ethylene glycol)- poly(ε-caprolactone); [d] crosslinked poly(ethylene glycol)-*b*-poly(2-methacrylate ethyl 5-hexynoicate); [e] hyaluronic acid surface-modified curcumin nanocrystals; [f] curcumin nanosuspension stabilized by mPEG2000-DSPE and soybean lecithin; [g] human serum albumin; [h] polymeric nanoparticles composed of N-isopropylacrylamide, vinylpyrrolidone, acrylic acid; [i] (methoxypolyethylene glycol)-poly (lactic-co-glycolic acid); [j] three-arm amphiphilic copolymer tricarballylic acid-poly (ϵ-caprolactone)-methoxypolyethylene glycol; [k] methoxy poly(ethylene oxide)-*b*-poly(ε-caprolactone); [l] nanoparticles based on chitosan hydrochloride/hyaluronic acid/polyethylene glycol; [m] poly(D,L-lactic acid)-glycerol; [n] poly(ε-caprolactone)-*b*-poly(N,N-diethylaminoethylmethacrylate)-r-poly(N-(3-sulfopropyl)-N-methacryloxyethy-N,N-diethylammoniumbetaine); [o] methoxy poly(ethylene glycol)-b-poly(δ-valerolactone); [p] solid lipid nanoparticles; [q] linear dendrimer-type methoxy-poly(ethylene glycol)-b-poly(ε-caprolactone); [r] poly(ε-caprolactone)-poly(ethylene glycol)-poly(ε-caprolactone)

The median and mean of the data for free curcumin and nano-encapsulated curcumin in rats are substantially different from each other. The mean is highly affected by two outliers (Figure 4A), whereas the median is less sensitive to such outliers. The reason for obtaining these high AUC values was not addressed by the authors [9, 14] in relation to the other reported data. As presented in the next section and Table S5, we propose that the difference is due to data miscalculation.

S3.3.1. Analysis of AUC outliers (references [9, 14])

Plasma concentration-time curves of curcumin nanoformulations and free curcumin were reproduced by extrapolating the data from the respective PK figures. The following studies [7, 13, 15, 16, 19, 31] were used to verify the analysis method. The AUC was calculated using PkSolver 2.0 add-in template in Microsoft Excel. Calculations were based on non-compartmental analysis. The calculation method yielded AUC values that were comparable to the reported AUC values for the validation studies (range AUC calculated:reported was 86-134%), whereas for the outlier studies there was a vast discrepancy between the reported AUC and the calculated AUC (range AUC calculated:reported was 0.05-0.16%).

Table S5. Comparison of reported AUCs and reanalyzed AUC value of free curcumin and curcumin nanoformulations using PkSolver. In the studies marked in orange, the reported and calculated AUC were comparable.

Dose [mg/kg]	Free curcumin			Curcumin nanoformulation			Ref.
	Reported AUC [μg•h/L]	Calculated AUC [μg•h/L]	AUC ratio calculated:reported [%]	Reported AUC [μg•h/L]	Calculated AUC [μg•h/L]	AUC ratio calculated:reported [%]	
10	474	425	90	622	662	106	[7]
25	29,894	33,041	111	148,079	159,542	108	[31]
15	967	1,130	117	4,464	3,860	86	[13]
2	6,708	6,370	95	8,964	10,705	119	[15]
10	1,705	1,717	101	7,672	8,288	108	[22]
10	2,298	2,745	119	5,761	7,742	134	[19]
15	668,415	577	0.09	2,772,523	2,187	0.08	[14]
10 / 5*	1,670,000	2,718	0.16	3,160,000	1,504	0.05	[9]

The validation studies are indicated in orange.
(*) the dose of free curcumin and curcumin nanoformulation was 10 and 5 mg/kg, respectively
Abbreviations: AUC, area under the curve; Ref., reference

S3.4. Physicochemical attributes of long-circulating curcumin nanoformulations

The delivery systems with a high NC:C AUC ratio (Table 2) exhibited different in vitro release profiles depending on the nature of the platform, such as the presence of pH-sensitive bonds or degradable polymers. Also, since the release medium and the setup of the release experiments were different between the studies, it is not possible to directly compare the results. Therefore, a standardized procedure using a suitable releasing medium that ensures sink condition is mandatory to perform and compare release studies of hydrophobic molecules like curcumin. A large volume of releasing medium or addition of surfactants or cosolvents are recommended strategies to maintain

sink conditions [42, 43]. Although these recommendations are not always followed, the general release profiles of curcumin nanoformulations with both marginal and high AUC ratios are comparable.

References

[1] D.A. Smith, K. Beaumont, T.S. Maurer, L. Di, Volume of distribution in drug design, J. Med. Chem., 58 (2015) 5691-5698.

[2] F. Gong, D. Chen, X. Teng, J. Ge, X. Ning, Y.L. Shen, J. Li, S. Wang, Curcumin-loaded blood-stable polymeric micelles for enhancing therapeutic effect on erythroleukemia, Mol. Pharmaceutics, 14 (2017) 2585-2594.

[3] P. Zou, L. Helson, A. Maitra, S.T. Stern, S.E. McNeil, Polymeric curcumin nanoparticle pharmacokinetics and metabolism in bile duct cannulated rats, Mol. Pharmaceutics, 10 (2013) 1977-1987.

[4] D. Sun, J.-K. Zhou, L. Zhao, Z.-Y. Zheng, J. Li, W. Pu, S. Liu, X.-S. Liu, S.-J. Liu, Y. Zheng, Y. Zhao, Y. Peng, Novel curcumin liposome modified with hyaluronan targeting CD44 plays an anti-leukemic role in acute myeloid leukemia in vitro and in vivo, ACS Appl. Mater. Interfaces, 9 (2017) 16857-16868.

[5] W. Wu, J. Wu, Q. Fu, C. Jin, F. Guo, Q. Yan, Q. Yang, D. Wu, Y. Yang, G. Yang, Elaboration and characterization of curcumin-loaded Tri-CL-mPEG three-arm copolymeric nanoparticles by a microchannel technology, Int. J. Nanomed. , 14 (2019) 4683-4695.

[6] D. Chen, J. Sun, In vitro and in vivo evaluation of PEG-conjugated ketal-based chitosan micelles as pH-sensitive carriers, Polym. Chem. , 6 (2015) 998-1004.

[7] Z. Song, R. Feng, M. Sun, C. Guo, Y. Gao, L. Li, G. Zhai, Curcumin-loaded PLGA-PEG-PLGA triblock copolymeric micelles: Preparation, pharmacokinetics and distribution in vivo, J. Colloid Interface Sci., 354 (2011) 116-123.

[8] M. Sun, Y. Gao, C. Guo, F. Cao, Z. Song, Y. Xi, A. Yu, A. Li, G. Zhai, Enhancement of transport of curcumin to brain in mice by poly(n-butylcyanoacrylate) nanoparticle, J. Nanopart. Res., 12 (2010) 3111-3122.

[9] Z. Ma, A. Shayeganpour, D.R. Brocks, A. Lavasanifar, J. Samuel, High-performance liquid chromatography analysis of curcumin in rat plasma: application to pharmacokinetics of polymeric micellar formulation of curcumin, Biomed. Chromatogr. , 21 (2007) 546-552.

[10] C. Yang, H. Chen, J. Zhao, X. Pang, Y. Xi, G. Zhai, Development of a folate-modified curcumin loaded micelle delivery system for cancer targeting, Colloids Surf., B, 121 (2014) 206-213.

[11] Z. Wu, M. Cai, X. Xie, L. He, L. Huang, Y. Chen, X. Luo, The effect of architecture/composition on the pH sensitive micelle properties and in vivo study of curcuminin-loaded micelles containing sulfobetaines, RSC Adv., 5 (2015) 106989-107000.

[12] J. Sun, C. Bi, H.M. Chan, S. Sun, Q. Zhang, Y. Zheng, Curcumin-loaded solid lipid nanoparticles have prolonged in vitro antitumour activity, cellular uptake and improved in vivo bioavailability, Colloids Surf., B, 111 (2013) 367-375.

[13] Z. Song, W. Zhu, J. Song, P. Wei, F. Yang, N. Liu, R. Feng, Linear-dendrimer type methoxy-poly (ethylene glycol)-b-poly (ε-caprolactone) copolymer micelles for the delivery of curcumin, Drug Deliv., 22 (2015) 58-68.

[14] R. Feng, Z. Song, G. Zhai, Preparation and in vivo pharmacokinetics of curcumin-loaded PCL-PEG-PCL triblock copolymeric nanoparticles, Int J Nanomedicine, 7 (2012) 4089-4098.

[15] S. Zhai, Y. Ma, Y. Chen, D. Li, J. Cao, Y. Liu, M. Cai, X. Xie, Y. Chen, X. Luo, Synthesis of an amphiphilic block copolymer containing zwitterionic sulfobetaine as a novel pH-sensitive drug carrier, Polym. Chem., 5 (2014) 1285-1297.

[16] Z. Song, W. Zhu, N. Liu, F. Yang, R. Feng, Linolenic acid-modified PEG-PCL micelles for curcumin delivery, Int. J. Pharm., 471 (2014) 312-321.

[17] X. Gao, F. Zheng, G. Guo, X. Liu, R. Fan, Z.-y. Qian, N. Huang, Y.-q. Wei, Improving the anti-colon cancer activity of curcumin with biodegradable nano-micelles, J. Mater. Chem. B, 1 (2013) 5778-5790.

[18] M. Gou, K. Men, H. Shi, M. Xiang, J. Zhang, J. Song, J. Long, Y. Wan, F. Luo, X. Zhao, Z. Qian, Curcumin-loaded biodegradable polymeric micelles for colon cancer therapy in vitro and in vivo, Nanoscale, 3 (2011) 1558-1567.

[19] Z. Song, Y. Lu, X. Zhang, H. Wang, J. Han, C. Dong, Novel curcumin-loaded human serum albumin nanoparticles surface functionalized with folate: characterization and in vitro/vivo evaluation, Drug Des., Dev. Ther., 10 (2016) 2643-2649.
[20] G. Lollo, G. Ullio-Gamboa, E. Fuentes, K. Matha, N. Lautram, J.-P. Benoit, In vitro anti-cancer activity and pharmacokinetic evaluation of curcumin-loaded lipid nanocapsules, Mater. Sci. Eng., C, 91 (2018) 859-867.
[21] S. Chen, Q. Li, H. Li, L. Yang, J.-Z. Yi, M. Xie, L.-M. Zhang, Long-circulating zein-polysulfobetaine conjugate-based nanocarriers for enhancing the stability and pharmacokinetics of curcumin, Mater. Sci. Eng., C, 109 (2020) 110636.
[22] J.Y. Hong, Y.Y. Liu, Y. Xiao, X.F. Yang, W.J. Su, M.Z. Zhang, Y.H. Liao, H.X. Kuang, X.T. Wang, High drug payload curcumin nanosuspensions stabilized by mPEG-DSPE and SPC: in vitro and in vivo evaluation, Drug Deliv., 24 (2017) 109-120.
[23] P. Ma, R.J. Mumper, Paclitaxel Nano-Delivery Systems: A Comprehensive Review, J Nanomed Nanotechnol, 4 (2013) 1000164.
[24] F. Wang, M. Porter, A. Konstantopoulos, P. Zhang, H. Cui, Preclinical development of drug delivery systems for paclitaxel-based cancer chemotherapy, Journal of Controlled Release, 267 (2017) 100-118.
[25] H. Gelderblom, J. Verweij, K. Nooter, A. Sparreboom, Cremophor EL: the drawbacks and advantages of vehicle selection for drug formulation, European journal of cancer (Oxford, England : 1990), 37 (2001) 1590-1598.
[26] S.T. Stern, M.N. Martinez, D.M. Stevens, When Is It Important to Measure Unbound Drug in Evaluating Nanomedicine Pharmacokinetics?, Drug Metabolism and Disposition, 44 (2016) 1934.
[27] Y. Liu, F. Chen, K. Zhang, Q. Wang, Y. Chen, X. Luo, pH-Responsive reversibly cross-linked micelles by phenol–yne click via curcumin as a drug delivery system in cancer chemotherapy, Journal of Materials Chemistry B, 7 (2019) 3884-3893.
[28] I.-S. Yoon, J.-H. Park, H.J. Kang, J.H. Choe, M.S. Goh, D.-D. Kim, H.-J. Cho, Poly(d,l-lactic acid)-glycerol-based nanoparticles for curcumin delivery, Int. J. Pharm. , 488 (2015) 70-77.
[29] Z. Song, W. Zhu, F. Yang, N. Liu, R. Feng, Preparation, characterization, in vitro release, and pharmacokinetic studies of curcumin-loaded mPEG–PVL nanoparticles, Polym. Bull., 72 (2015) 75-91.
[30] Y. Xu, S. Asghar, L. Yang, Z. Chen, H. Li, W. Shi, Y. Li, Q. Shi, Q. Ping, Y. Xiao, Nanoparticles based on chitosan hydrochloride/hyaluronic acid/PEG containing curcumin: In vitro evaluation and pharmacokinetics in rats, Int. J. Biol. Macromol. , 102 (2017) 1083-1091.
[31] C. Gong, S. Deng, Q. Wu, M. Xiang, X. Wei, L. Li, X. Gao, B. Wang, L. Sun, Y. Chen, Y. Li, L. Liu, Z. Qian, Y. Wei, Improving antiangiogenesis and anti-tumor activity of curcumin by biodegradable polymeric micelles, Biomaterials, 34 (2013) 1413-1432.
[32] Y. Yu, X. Zhang, L. Qiu, The anti-tumor efficacy of curcumin when delivered by size/charge-changing multistage polymeric micelles based on amphiphilic poly(beta-amino ester) derivates, Biomaterials, 35 (2014) 3467-3479.
[33] K. Jiang, M. Shen, W. Xu, Arginine, glycine, aspartic acid peptide-modified paclitaxel and curcumin co-loaded liposome for the treatment of lung cancer: in vitro/vivo evaluation, Int J Nanomedicine, 13 (2018) 2561-2569.
[34] P. Ji, L. Wang, Y.W. Chen, S.Q. Wang, Z.H. Wu, X.L. Qi, Hyaluronic acid hydrophilic surface rehabilitating curcumin nanocrystals for targeted breast cancer treatment with prolonged biodistribution, Biomater. Sci., 8 (2020) 462-472.
[35] Y.Z. Hu, Y.H. He, J.R. Ji, S.P. Zheng, Y.Z. Cheng, Tumor targeted curcumin delivery by folate-modified mPEG-PCL self-assembly micelles for colorectal cancer therapy, Int J Nanomedicine, 15 (2020) 1239-1252.
[36] L. Li, D. Xiang, S. Shigdar, W. Yang, Q. Li, J. Lin, K. Liu, W. Duan, Epithelial cell adhesion molecule aptamer functionalized PLGA-lecithin-curcumin-PEG nanoparticles for targeted drug delivery to human colorectal adenocarcinoma cells, Int. J. Nanomed. , 9 (2014) 1083-1096.

[37] D. Duan, A. Wang, L. Ni, L. Zhang, X. Yan, Y. Jiang, H. Mu, Z. Wu, K. Sun, Y. Li, Trastuzumab- and Fab' fragment-modified curcumin PEG-PLGA nanoparticles: preparation and evaluation in vitro and in vivo, Int J Nanomedicine, 13 (2018) 1831-1840.
[38] P. Colucci, J. Turgeon, M.P. Ducharme, How critical is the duration of the sampling scheme for the determination of half-life, characterization of exposure and assessment of bioequivalence?, J. Pharm. Pharm. Sci., 14 (2011) 217-226.
[39] R. Jamwal, Bioavailable curcumin formulations: A review of pharmacokinetic studies in healthy volunteers, J. Integr. Med, 16 (2018) 367-374.
[40] M. Bagheri, M.H. Fens, T.G. Kleijn, R.B. Capomaccio, D. Mehn, P.M. Krawczyk, E.M. Scutigliani, A. Gurinov, M. Baldus, N.C.H. van Kronenburg, R.J. Kok, M. Heger, C.F. van Nostrum, W.E. Hennink, In Vitro and In Vivo Studies on HPMA-Based Polymeric Micelles Loaded with Curcumin, Molecular Pharmaceutics, 18 (2021) 1247-1263.
[41] J. Duan, Y. Zhang, S. Han, Y. Chen, B. Li, M. Liao, W. Chen, X. Deng, J. Zhao, B. Huang, Synthesis and in vitro/in vivo anti-cancer evaluation of curcumin-loaded chitosan/poly(butyl cyanoacrylate) nanoparticles, Int. J. Pharm., 400 (2010) 211-220.
[42] S.A. Abouelmagd, B. Sun, A.C. Chang, Y.J. Ku, Y. Yeo, Release kinetics study of poorly water-soluble drugs from nanoparticles: Are we doing it right?, Mol. Pharmaceutics, 12 (2015) 997-1003.
[43] M. Sheybanifard, N. Beztsinna, M. Bagheri, E. Miriam Buhl, J. Bresseleers, A. Varela-Moreira, Y. Shi, C.F. van Nostrum, G. van der Pluijm, G. Storm, W.E. Hennink, T. Lammers, J.M. Metselaar, Systematic evaluation of design features enables efficient selection of Π electron-stabilized polymeric micelles, Int. J. Pharm., (2020) 119409.
